# An analysis of the leverage effect of “Internet +” on the economic spillover of sports industry

**DOI:** 10.1371/journal.pone.0288778

**Published:** 2023-07-19

**Authors:** Cuixia Yi, Yang Tao, Fu Bo, Liu Zhipeng, Dong Yu, Liu Lingzhi

**Affiliations:** 1 School of Humanities and Social Science, Xi’an Jiaotong University, Xi’an, Shaanxi, China; 2 Physical Education School, Shaanxi Normal University, Xi’an, Shaanxi, China; 3 Xi’an Physical Education University, Xi’an, Shaanxi, China; 4 Graduate Department, Shanghai University of Sport, Shanghai, China; 5 Xi’an Medical University, Xi’an, Shaanxi, China; 6 School of Public Administration, Xi’an University of Finance and Economics, Xi’an, Shaanxi, China; Bahria University, PAKISTAN

## Abstract

Given the unprecedented and profound impact on the traditional sports industry and its economic driving force brought by the “Internet +”, the purpose of this study is to explore the driving relationship between the sports industry and local economic development and deconstruct the dynamic regulation mechanism of “Internet +” on the economic spillover of the sports industry. Empirical data collected from China’s 11 inter-provincial panel data from 2015–2019 were analyzed through the fixed-effects and threshold models. The study finds that the economic spillover of the sports industry has significantly promoted regional economic development, and the regulation effect of the Internet has complex non-linear characteristics. Only when Internet development exceeds a specific threshold can it develop a benign complementary mechanism with the sports industry. The impact of “Internet +” on the threshold regulation of the sports industry economic spillover has spatial and temporal differentiation characteristics in the three dimensions of Internet hardware level, Internet penetration rate, and Internet application value. The results of this study extend the current study, indicating that it is a practical choice for local governments to develop the economy through the sports industry. Provinces and cities should enhance the Internet penetration rate, expand Internet business applications, and upgrade Internet development to a specific point to maximize the drive of positive spillover. Regional heterogeneity requires differential regulation strategies and more concentration on the middle and western regions.

## Introduction

Entering the new era, the role of China’s sports industry in national economic and social development has become increasingly prominent [1], which has evolved a new growth point of the national economy, and the sports industry plays a highly significant role in facilitating the construction of economic power, the construction of Healthy China and the construction of a sports power [2]. At the third session of the 12th National People’s Congress, the “Internet +” action program was first proposed by Premier Li Keqiang in the government work report [3]. The sports industry in China maintained torrid growth during 2016–2019, as the total scale increased beyond a trillion yuan, up by 55.1%, which is 1.14% of GDP surged from 0.87%.

Due to the Covid-19 pandemic, the speed of economic growth of the national value-added of the tertiary industry slowed sharply in 2020 [4]. While the sports industry transformed into digital sports with the help of new technologies, such as big data [5], “Internet +” [6], 5G [7], etc., the value-added of sports media and information service industry maintained 18.9% growth, and the value-added of physical education and training industry expanded 5.7% through new business models like live-streaming fitness [8] and online training [9]. The downward pressure on China’s economic growth is rising continuously, and the increasing trend of the sports industry can not only hedge the risk to some extent but also contribute to industrial restructuring. Therefore, this study concentrates on analyzing the relationship between the sports industry and regional economic development and how to maximize the spillover effect of the sports industry to promote the regional economy’s development better.

In 2021, the state sports general administration of China officially released “the 14th Five-Year Plan for Sports Development”, which pointed out the implementation of the digital strategy of the sports industry, the promotion of the “Internet +” by adhering to both sides of supply and demand, and the promotion of the high-quality development of the sports industry. As sports have been taken into the digital era by “Internet +”, traditional sports are confronted with tremendous impact, which includes positive impacts like the Internet dissemination of sports [10], Internet sports esports rapid popularity [11], and negative impacts, for instance, Internet sports enterprises squeeze offline entities’ living space [12]. Youth indulge in esports which is not conducive as it occupies the time for outdoor recreation [13]. The positive view reckons that “Internet +” can accelerate the expansion of the sports industry [14, 15] and amplify new economic growth space [16], while the negative view is that “Internet +” releases more virtual economy, which will cause a “crowding out effect” on the traditional sports industry and inhibit the economic driving effect of the sports industry. Consequently, there are two reverse paradoxes: “promotion theory” and “inhibition theory” of the economic spillover effect of “Internet +” on the sports industry.

The Internet has entered all circles of people’s lives, but its impact on the economic spillover effect of the sports industry is still uncertain. Therefore, how to stimulate the economic spillover effect of the sports industry in the context of the Internet is of great theoretical value and practical significance. This study concentrates on the economic spillover effect of the sports industry and utilizes China’s inter-provincial panel data from 2015–2019 to deconstruct the mechanism of the Internet. The purpose of this study was to: (1) explore the leverage effect of economic spillover of the sports industry in the context of “Internet +”; (2) examine what the specific regulation mechanism is; (3) investigate the practical needs of transformation of the sports industry and the dilemmas during the process of transformation. This study offers new insights into the role of the Internet in the process of the sports industry driving the regional economy and suggests future directions for sports industry research and government policies.

A linear estimation model with sports industry economic spillover as the dependent variable was designed based on the previous studies. The level of “Internet +” development was employed as the threshold regulation variable to examine its dynamic impact on the economic spillover of the sports industry. Inter-provincial panel data from 11 provinces and regions from 2015–2019 were collected and analyzed through the fixed-effects model, threshold model, robustness test, and heterogeneity analysis. This study enhances the current research by analyzing the sports economic spillover on the regional economy under the influence of the Internet. It supports the findings of Ruan’s study [17] and extends the theoretical study of Huang et al. [18] by incorporating the Internet to explain the sports spillover effects on the regional economy through empirical analysis. Moreover, this study also validates the results of Jiang’s research [19] on the sports industry and regional economy heterogeneity.

The study finds a significant correlation between the sports industry and the regional economy. A positive relationship of dependence between the impact of the sports industry on regional economic development and the regulation mechanism of the Internet is significant, but the regulating effect of the Internet on the economic spillover of the sports industry has complex non-linear characteristics. Only when the level of Internet development exceeds a specific threshold can it develop a benign complementary mechanism with the sports industry. Based on the empirical results, this study suggests that it is a practical choice for local governments to develop the economy through the sports industry. As the “Internet +” is the binding force for transforming and upgrading the sports industry, provinces, and cities must enhance the Internet penetration rate, expand Internet business applications, and upgrade the level of Internet development to a specific point to maximize the drive of positive spillover. The regional heterogeneity nature requires more concentration on the middle and western regions.

## Literature review

### The impact of sports industry on regional economic development

Some local political and community leaders, and the owners of professional sports teams frequently claim that professional sports facilities and franchises are important engines of economic development in urban area [20]. Mega sports events or small-scale sports events as long as they are organized and controlled locally would grow in relevance as a way to promote local and regional economic development [21]. Gibson, Willming, and Holdnak noted that Mega sports events may generate more positive effects for host communities, as their control remains localize and any economic benefits often stay within host communities [22]. In China, since the State Council issued "Several Opinions on Accelerating the Development of Sports Industry and Promoting Sports Consumption" in 2014, China’s sports industry has entered a new era. The nominal annual average growth rate of the total output value of the sports industry during the four years from 2016 to 2019 was much higher than the nominal growth rate of the national GDP. As a result, the proportion of the total output value of the sports industry to GDP gradually increased and became a new growth point of the national economy. The relationship between the sports industry and economic development gradually became a hot issue for research in sports academia. As an industrial sector covering manufacturing and service industries, the sports industry is an integral part of regional economic growth, affecting the total value of the regional economy, employment issues, economic structure, and tax status [23, 24].

Among the studies on the driving effect of sports industry on regional economic development, Huang noted that “Olympic Economy” that formed by sports industry will have an important role in promoting China’s economic development [25]. Chen Wensheng’s empirical analysis of the contribution of the sports industry to economic growth shows that each percentage point increase in investment in the sports industry can boost the national economy by 0.07 percentage points [26]. As a new regional economic growth point, the sports industry has become a pillar industry of the regional economy and is of great significance in promoting economic transformation and upgrading [27]. The sports industry and regional sustainable development in eastern China are showing a steady growth trend, and the sports industry is growing faster than regional sustainable development [28]. With the increasing demand for sports in China and the improving quality of the sports industry, the collective economic effect of industrial linkages will become more prominent and the industrial structure within the region more rational, thus promoting the shift of resources to high productivity areas [19]. The sports industry not only contributes to transforming economic development types but also helps adjust the industrial structure, expand domestic demand, increase employment opportunities, improve labor efficiency, and reduce medical expenses [18]. In the study on the interaction between the sports industry and regional economic development, Zhang Liang noted that when the per capita disposable income (PCDI) of urban residents is less than 13659.26 yuan, increasing investment in the sports industry is not conducive to regional economic growth; in areas where the PCDI is between 13659.26 and 21168.79 yuan, the effect of investment in the sports industry on regional economic growth is not significant; when the PCDI exceeds 21168.79 yuan, increasing investment will significantly contribute to regional economic development [29]. Economic growth, sports industry types, the degree of integration between the sports industry and economic development, sports industry action time, and sports industry scale are the main factors affecting the sports industry and promoting the development of the sports economy [30].

The added value of the sports industry is an important indicator that can reflect the development level of the sports industry. For example, from the data of 2017, the added value of the sports industry in Fujian, Guangdong, and Jiangsu in the eastern region is 132.401 billion yuan, 132.186 billion yuan, and 121.955 billion yuan, respectively. At the same time, the added value of the sports industry in Henan, Hunan, and Anhui in the central region reveals that the level of regional economic development not only directly affects the results but also determines the ability of their sports industry development [31]. The coupling degree between the sports industry and economic development in Shanghai in the eastern region was 0.4986, 0.4965, and 0.4992 from 2015 to 2017, respectively, and the coupling degree between the sports industry and economic development in other provinces during the same period was no higher than 0.5. The results reflect that the sports industry has been improving its quality and efficiency in recent years, with an average annual growth rate higher than that of GDP in the same period. However, the regional economy and sports industry system is a contradictory and unified; the two complement each other and promote each other to produce a combined effect, achieving sustainable and joint development [32].

### The impact of “Internet +” on the sports industry

Social Media Use in 2021, which was published on April 7, 2021, reported that a majority of Americans say they use YouTube and Facebook, and A great number of Facebook, Snapchat and Instagram users say they visit these platforms on a daily basis, Seven-in-ten Facebook users say they use the site daily, including 49% who say they use the site several times a day [33]. Organizations are using internet marketing for sports, for instance, ticket promotions, scores and stats, and schedules and information [34]. The Internet has been deeply integrated into all corners of sports, and industrial networking has become an inevitable development tendency in all fields of society at present. The “Internet +” sports industry is a new format developed using modern Internet technology [35], cloud computing technology [36], Internet of things technology [37], and other advanced science and technologies to integrate with the traditional sports industry [38]. By applying “internet+” to the sports industry, the innovative development of new formats such as Internet broadcasting, intelligent software and hardware, e-commerce, and esports can be realized. For instance, the revenue of sports software has increased from 108 million yuan in 2010 to 352 million yuan in 2021 [39]. “Internet +” utilizes data on the preferences of the public to ensure a stable and balanced relationship between supply and demand and to maximize the quality and efficiency of the use of sports resources [40]. As the Internet promotes the sports industry’s development, it also poses certain challenges, and we may be struggle to understand and unleash the full beneficial impact of digital technologies due to a lack of know-how and missing resources [41].

The “Internet +” is affecting the business market. The Internet has extended the resource boundary of the sports industry from the “Supply-side” to the “Demand-side”, and the transformation of the business structure from the B2C model to the C2B model has forced the production system to change, and the consumption mode of consumers has changed from direct purchase to the rental form based on the sharing model, and the production of sports goods has also changed from homogeneity and standardization to personalization, and the ability to accurately understand the personality of consumers, rapidly respond and combine production has become the key to product competitiveness [42]. The transformation of the sales of sports products in the context of “Internet +” has revolutionized the process of sports consumption, with material consumption such as sports equipment and apparel, and service consumption such as sports events and training changing to O2O (Online to Offline) mode, meanwhile, “Internet +” has also profoundly affected the consumption style and process of products including sports lottery, esports, sports copyright, physical education, and scientific research, and culture [43], for instance, according to the data of Taiwan Sports Lottery from May 1, 2018 to April 30, 2019, the betting rate with mobile phone and tablet computer was as high as 70% [44].

The shift of consumption characteristics in the context of “Internet +” has reconstructed the form of economic rents obtained by enterprises, and in the age of Internet 2.0, the sharing model has become prevailing. The connecting dividend rental has gradually replaced the ability rental under the role of the Internet so that enterprises no longer make money by directly selling products, nor do they concentrate on chasing product sales dividends. Thus a great number of traditional fitness companies have already started to utilize their offline resources to enhance digital upgrades [45]. The shift was even more prominent after the Coivd-19 pandemic. An analysis of 251 people revealed that 67.7% of respondents were not interested in visiting offline retail channels in the future [46]. Under the circumstance of “Internet +”, the cost of additional display and space of goods on the platform is almost zero, and the homogenization of products is severe, resulting in oversupply. Enterprises will usually adopt the method of cutting prices to enhance profits to occupy a particular market share, which will trigger vicious competition in the Internet plus sports tourism industry. The “Internet +” affects the entire sports industry ecology. The new business model of “Internet +Sports” still has many potential challenges due to its short development history, single industrial structure, and immature operation system. From the internal aspect, the sports industry has developed a new profitable model with the support of Internet technology, but it still counts on the joint completion of advertising investment and product sales, thereby not substantially changing the profit model of sports products. From the external aspect, the “Internet +” sports industry involves many industry fields and social sectors such as hardware and software and other network technologies, media platforms, e-commerce logistics, etc. This has regulated and restrained the sports industry by many government departments, and the unclear division of functions and management attribution has prevented the sports industry from getting sufficient space for development [47]. Moreover, the threat of network and information security faced by the sports industry in the process of transformation based on digital technology is worsening [48]. The policy guidance of the sports industry under the background of “Internet +” is not precise and refined enough, and the cognition of the development and upgrading of China’s sports industry remains to be improved [49].

### Commentary

As seen in Table 1, most Chinese scholars stated that the sports industry could assist in developing the regional economy. Some studies use the same database as this paper to analyze the correlation between the sports industry and regional economy and put forward the concepts of regional heterogeneity [19], coupling degree [28], and evaluation index [32], which provide rich research ideas and a theoretical basis for the study of the sports industry and regional economy. For instance, a time-fixed effects model with the scale and value-added of regional sports industry output as the independent variable was applied to examine the economic growth and sports industry structure in 12 provinces and cities in different country regions. Controlling for other variables, the total output and value-added of the sports industry are significant at the 1% level and contribute significantly to regional GDP [19]. Besides, Sun investigated the mechanism of sports consumption boosting urban economic development through panel data of major cities in Jiangsu Province, using the level of sports consumption as the core explanatory variable [50]. The multi-dimensional investigation is combined with the industrial characteristics and geographical location, which provides a wealth of research ideas and theoretical foundations for the research on the sports industry and regional economy. However, it can be seen that there exist specific differences in the industrial development stage, industrial pattern, and market environment in China’s sports industry in different regions. Therefore, the results from a nationwide study can not fully represent the relationship between the sports industry and the regional economy.

**Table 1:** Different research on sports and regional economy.

Region/Scope	Context	Research Methods	Research Subjects
National	None	Quantitative	Core elements for coordinated development of regional economy and sports industry [27].
12 Provinces and Cities	None	Quantitative	The coupling degree and coordination degree of sports industry and regional economy [28].
National	China’s New Era	Qualitative	The strategy of the sports industry contributes to the construction of economic power. The sports industry is conductive to the ecological economy of regional development [18].
12 Provinces and Cities	None	Quantitative	The relationship and heterogeneity of sports industry and regional economy [19].
12 Provinces and Cities	None	Quantitative	Regional characteristics of the sports industry development [31].
14 Provinces and Cities	Digital Economy	Quantitative	the Impact of Digital Economy on the High-Quality Development of Sports Industry [17].
13 Cites in Jiangsu Province	New Development Paradigm	Quantitative	the impact of the sports consumption on urban economic development [50]
National	Health China	Qualitative	the development of the Internet plus sports [51]

In addition, in the background of industrial networking, domestic researchers have focused on the prospect and strategy of sports industry development under the context of “Internet +”, and a comprehensive analysis reveals that “Internet +” influences not only the sports business market but also the whole sports industry ecology. For example, Ruan’s study employed a random effects model with the level of development of the digital economy as the explanatory variable to investigate its effect on the structure of the sports industry [17]. However, the present studies are mainly based on qualitative theoretical deduction research [18, 51], which lacks objective quantitative research support, thus hindering the expansion of research profundity to a certain extent. This paper intends to analyze the driving relationship between the sports industry and regional economic development, explore the profound and intrinsic attributes of the sports industry in the context of “Internet +”, and deconstruct the dynamic regulation mechanism of “Internet +” on the economic spillover of the sports industry by applying statistics and econometric model, so as to promote the integration and drive of “Internet +” and digital sports, and empower high-quality development in China.

## Materials and methods

### Theoretical assumptions

The development of the sports industry has many influences on regional economic development, and the “Internet +” has had a disruptive impact on the sports industry, for instance, what kind of ripple effects will the “Internet +” have on the economic spillover of the sports industry? The empirical understanding is that the Internet medium and mobile communication technology are evolving rapidly, the form of sports and entertainment activities are becoming increasingly abundant, and the market space released by the Internet event broadcasting is more extensive than the traditional TV medium, boosting the modernization of the sports industry and accelerating the popularity of sports audiences, which will expand the scale effect of the sports industry and accelerate the economic spillover. In developed Western countries, the sports industry has become essential to social and economic development precisely in the era of communication innovation, especially after the per capita GDP exceeded the US $10,000; the sports industry and the commercial economy have merged to form an economic driver of enormous scale. The commercial revenue from advertising of some sports events even exceeds the revenue from game viewing itself, and the proportion in the consumption structure has been rising, boosting the rapid expansion of upstream and downstream industries.

In contrast, the impact of the “Internet +” on the traditional sports industry has continued to expand, including the massive growth of cultural and entertainment content on the Internet and the overwhelming encroachment of esports and Internet games on the leisure time of juveniles, which is likely to cause a “crowding-out effect” on the development of the sports industry and inhibit the economic spillover from the sports industry. According to the relevant statistics, the time spent on sports activities by Chinese youths aged 6–18 has been decreasing year by year, whereas the time spent on Internet games has been continuously rising. Despite the increasing prosperity of “Internet” culture and entertainment, if increasingly more spare time is spent on the “Internet” virtual economy, the target audience focusing on and participating in sports activities will become dwindling, which will not be beneficial to the physical and mental growth of youths as well as negatively affect the high-quality economic development in the long run.

From this, it appears that the ripple effect of “Internet +” on the economic spillover of the sports industry is not one-way beneficial or inhibiting. The above paradox leads to the core theoretical hypothesis of this paper: The impact of “Internet +” on the economic spillover of the sports industry is sophisticated, and there are two potential “crowding-in effect” and “crowding-out effect” with specific symbiotic evolutionary laws. Therefore, in the following section, we designed an econometric model with the sports industry development as the explanatory variable and the Internet development as the regulation variable to test the dynamic impact of “Internet +” on the economic spillover of the sports industry through empirical research.

### Econometric model

#### Estimation model of the spillover impact of sports industry on economic development

Using the level of sports industry development (Sport) as the explanatory variable and the level of regional economic development (GDP) as the explained variable, a linear estimation model is designed to test the linear spillover impact of the sports industry on economic development.


$$GDP_{it}=\alpha Sport_{it}+\theta_{n}C_{\mathrm{it}}+\mu_{i}+v_{t}+\varepsilon_{it}$$ (1)

*α* is the elasticity coefficient of the impact of the sports industry on the level of economic development, *C*_*it*_ A represents each control variable for reducing endogenous disturbances, *θ*_*n*_ is the control variable affecting the elasticity coefficient, *i* represents the provinces, *t* indicates different years, *m*_*i*_ denotes the individual effect of each sample that does not vary with time, *v*_t_ is the time section effect, and error term is $\varepsilon_{it}\sim iid(0,\sigma^{2})$.

#### The dynamic regulation model of economic spillover from “Internet +” to the sports industry

In this paper, we applied Hanse’s panel threshold regression model. Previous research utilized group testing or constructed multiplicative estimation models to analyze nonlinear threshold characteristics. However, both methods have certain shortcomings. First, it is difficult to solidify the grouping criteria in group testing, which may distort threshold estimation and lack of corresponding significance test. The multiplicative estimation model can estimate the overall impact threshold, but the cross-term cannot effectively analyze the impact relationship between core explanatory variables and threshold variables. The estimation results have limitations in interpretation. Hansen’s panel threshold regression model effectively compensates for the technical shortcomings of the above two threshold tests by constructing piecewise functions of the regression coefficients of the explanatory variables, which can not only estimate the thresholds but also further test the reasonableness of the thresholds for significance. The model captures this nonlinear relationship when there is a threshold effect, i.e., when the relationship between the explanatory and explained variables changes significantly at a threshold point. The data utilized in the study are rather balanced and stable, and the sample size is relatively large, while this contributes to a more accurate estimation of the threshold and related parameters. Also, one of the purposes of this study was to investigate the threshold regulation effect of the Internet on the economic spillover of the sports industry. Therefore, this model fits the purpose and data requirements of this study.

Using Internet development level Internet as a threshold moderating variable, *η* is the threshold. The level of sports industry development is taken as the explanatory variable and the level of regional economic development is used as the explained variable. We obtain the dynamic regulation impact estimation model of “Internet +” on the economic spillover of the sports industry:

$$\begin{array}{l} GDP_{it}=\alpha_{1}Sport_{it}•I(Internet_{it}\leq \eta_{1})+\alpha_{2}Sport_{it}•I(Internet_{it}>\eta_{1})+\ldots +\\ \alpha_{2n-}{}_{1}Sport_{it}•I(Internet_{it}\leq \eta_{n})+\alpha_{2n}Sport_{it}•I(Internet_{it}>\eta_{n})+\theta_{n}C_{\mathrm{it}}+\mu_{i}+v_{t}+\varepsilon_{it} \end{array}$$ (2)

In the above equation, *η*_*n*_ is the threshold that assumes a change in the level of Internet development. *α*_*n*_ is the elasticity coefficient of the effect of the level of Internet development on the level of production technology when the level of Internet development is in different intervals. *I*(·) represents the indicator function that tests the hypothesis of the existence of a threshold for the level of Internet development. When the conditions in brackets are matched, the threshold assumption is qualified to take the value of 1, and vice-versa to take the value of 0. The repeated iterative tests to derive the threshold *η*_*n*_ and the elasticity coefficient of nonlinear influence *α*_*n*_, and Non-linear impact trajectory of the sports industry on the economic development level is carved.

### Measurement of variables

#### Explained variables

GDP, as an important evaluation index of macroeconomic output, can reflect the fundamentals of economic development in different regions, and the GDP growth rate is chosen to reflect the economic development level of different regions in the model regression, as shown in Table 2.

**Table 2:** Variable operationalization.

Variables	Operation Definition	Indicator	Measure
GDP	the economic development level of the regions	GDP growth rate	Ratio
Sport	the level of sports industry development	the scale of regional sports industry output value	Ratio
Internet-1	Internet construction level	the distance of Internet lines laid in each province and region	Ratio
Internet-2	Internet penetration rate	the total number of Internet users in each region	Ratio
Internet-3	Internet commercial application value	commercial transaction amount of regional Internet data flow	Ratio
Urbanization	the level of urbanization	the proportion of the urban population	Ratio
Human	human resources conditions	the number of years of education per capital in the region	Ratio
Trade	the contribution of the trade	the ratio of import and export volume to GDP in the region	Ratio
Capital	the total productive capital owned by an economy	region’s capital stock in the current year	Ratio

#### Explanatory variables

The sports industry is an essential part of national economic development. To assess the level of the sports industry development (Sport), we take the scale of regional sports industry output value as the evaluation criterion and do logarithmic treatment in the regression to reduce the influence of variance, as displayed in Table 2.

#### Threshold regulation variables

The level of “Internet +” development (Internet). In order to comprehensively reflect the development level of “Internet +”, we referred to Han’s [52] research method and decomposed the Internet development level into Internet construction level (Internet-1), which is measured by the distance of Internet lines laid in each province and region; Internet penetration rate (Internet-2), which is reflected by the total number of Internet users in each region; and Internet commercial application value (Internet-3), which is expressed by the commercial transaction amount of regional Internet data flow (Table 2).

#### Control variables

The selection of control variables should be based on theoretical foundations and previous studies, and those variables that may interfere with the correlation between explanatory and explained variables should be selected. According to the current literature, urbanization [53], trade [54], human capital [55], and capital stock [56] may affect the correlation between the explanatory and the explained variables. Therefore, to achieve unbiased test results and consider the measurement and operationalization of the variables, this study includes relevant control variables affecting economic development in the model. Specifically, these include: Urbanization, measured by the proportion of the urban population in the region; humans, reflected by the number of years of education per capita in the region; Trade, measured by the ratio of import and export volume to GDP in the region; and Capital is evaluated by the region’s capital stock in the current year and treated as a logarithm in the regression (Table 2).

### Data sources and testing

In 2014, the General Office of the State Council issued the Opinions on Promoting National Fitness, Sports Consumption, and High-Quality Development of the Sports Industry. Since then, China’s sports industry’s added value and growth rate have grown significantly. However, the rapid development of the sports industry was hampered by Coivd-19 pandemic in 2020. Therefore, this paper selects the research period from 2015–2019, which is more representative; considering the consistency of relevant statistical standards and the availability of data, the sample includes 11 provinces and regions (Descriptive statistics are shown in Table 3 below). The data sources are mainly from the China Internet Statistics Report, China Statistical Yearbook, China Sports Statistical Yearbook, provincial and regional sports development reports, etc. To predict the time tendency disturbance of the macro data, we did a unit root test, and the results suggested that the data were first-order stationary. Meanwhile, the Kao test found that there was a stable equilibrium stable relationship between the core variables, which met the model regression requirements.

**Table 3:** Descriptive statistics of variables.

Variables	Number	Max	Min	Mean	St-deviation	Stationary Test
GDP	55	11.587	6.814	10.395	0.552	△
Sport	55	8.595	4.048	6.973	6.814	△
Internet-1	55	1.251	0.340	0.530	0.218	△
Internet-2	55	14.921	12.918	14.111	0.543	△
Internet-3	55	8.962	5.818	6.764	0.717	△
Urbanization	55	0.896	0.400	0.550	0.120	△
Human	55	11.455	7.781	9.022	0.752	△
Trade	55	1.021	0.040	0.144	0.240	△
Capital	55	10.824	8.476	9.707	0.617	△

^a^These 11 provinces and regions are Shanghai, Shandong, Zhejiang, Hebei, Guangdong, Anhui, Chongqing, Hunan, Sichuan, Guizhou, and Jiangxi.

## Results

### Linear driving relationship between sports industry development level and regional development economic level

Three models are usually available for panel data analysis: the mixed estimation model, fixed effects model, and random effects model. However, the differences in time and cross-sectional dimensions of the data in this paper are relatively slight, and the p-value of the Hausman test is not significant, i.e., an individual fixed effect exists. Therefore, this study applied the fixed-effects model to assess the linear relationship between the level of sports industry development and regional economic development. The results indicated (see model L1 in Table 4) that the elasticity of influence between the level of sports industry development (Sport) and the level of regional economic development (GDP) was 0.010, which also passed the 10% significance level test, reflecting the positive facilitation relationship between sports industry and regional economic development. This is coherent with the empirical hypothesis that the modernized the sports industry contains an immense commercial value, and the marginal consumption generated by the sports industry has a particular promotion effect on economic development. At the same time, the significant scale effect of the sports industry drives the dramatic expansion of upstream and downstream industries, providing new economic growth points for regional economic development and becoming an essential pillar of China’s economic transformation.

**Table 4:** The linear drive of the sports industry to economic development.

Variables	FE/GDP Model-L1	FE/GDP Model-L2	FE/GDP Model-L3	FE/GDP Model-L4	FE/GDP Model-L5	RE/GDP Model-L6
Sport	0.010* (1.88)	0.017 (0.60)	0.014 (1.65)	0.008 (0.74)	0.013 (1.45)	0.009 (0.48)
Urbanization	-1.111** (-2.29)		5.395*** (7.03)	4.691*** (4.84)	4.607*** (4.64)	0.381 (0.49)
Human	-0.009 (-0.18)			0.275* (1.83)	0.242* (2.01)	0.043 (0.40)
Trade	-0.045 (-1.37)				-0.114 (-0.54)	-0.057 (-0.39)
Capital	1.134*** (11.05)					0.826*** (7.45)

^a^*, **, *** indicate 10%, 5%, and 1% significant levels, respectively.

The estimation results of the control variables suggest that the level of urbanization development (Urbanization) has a slightly negative effect on regional economic development, which could be attributed to the fact that China’s urbanization level is already relatively high. The development trend appears to be marginal decreasing, which is not conducive to the stability and sustainability of economic growth. The impact of human capital conditions (Human) on regional economic development is not statistically significant, and the impact on economic growth has gradually weakened in recent years due to the increase in the level of education in the country and the slowdown in the growth rate of the average age of receiving education. Trade openness (Trade) is ineffective in stimulating regional economic development, demonstrating that the trade structure and product value chain still need to be upgraded, and the efficiency of economic spillovers driven by low-value-added products is low. Capital (Capital) has a noticeable positive impact on regional economic development, indicating that capital investment leverages the transformation and upgrading of the sports industry, consequently driving regional economic development.

### Iterative spillover impact of “Internet +” on economic spillover of sports industry

In this paper, the level of Internet development is analyzed from three aspects, using the level of infrastructure (Internet-1), the penetration rate (Internet-2), and the value of development and application (Internet-3) as threshold regulation variables to study the iterative impact of “Internet +” on the economic spillover of the sports industry, comprehensively considering the impact of the level of Internet development on the results of the econometric model.

Firstly, the level of the Internet infrastructure (Internet-1) is used as the threshold regulation variable, the level of sports industry development is used as the dynamic explanatory variable, and the level of regional economic development is considered as the explained variable. The iterative effect of the level of Internet infrastructure on the economic spillover of the sports industry is examined, with the results presenting a comparatively complex dynamic moderating relationship (model T1- in Table 5). When the level of Internet infrastructure is in the first threshold range [0 0.6794], the impact of the sports industry development level on regional economic development is significantly negative, which shows an inhibitory relationship; however, when the level of Internet infrastructure is raised to the second threshold range [0.6794 0.8379], the impact of sports industry development level on regional economic development becomes positive and insignificant; when the level of Internet infrastructure construction level further increases to the third threshold interval [0.8379 0.8790], the economic spillover impact of the sports industry returns to negative and insignificant. When the level of Internet infrastructure exceeds 0.8790, the elasticity coefficient of the impact of the level of sports industry development on the regional economy is 1.0199 and passes the 1% significance level test, effectively driving economic spillover from the sports industry. As a result, there is a symbiotic evolutionary mechanism of “crowding-in effect” and “crowding-out effect” for “Internet +” sports industry economic spillover. Despite the inhibiting influence on the economic development of the sports industry at the preliminary stage of Internet construction, the traditional sports industry can realize co-rail spillover and stimulate the economic vitality of the sports industry through the integration and upgrading with the emerging “Internet +” industry to create a new business model of sports industry at a specific intensity, which is in conform to the theoretical mechanism hypothesis.

**Table 5:** Threshold estimation and test results for the impact of “Internet +” regulation.

Threshold model	Threshold test	Estimated	F	P	BS Frequency
Internet-1 the threshold regulation model T1-1	Single Threshold	0.6794	7.8718***	0.010	500
	Dual Threshold	0.8379	9.3459***	0.000	500
	Triple Threshold	0.8790	12.002***	0.004	500
Endogenous test model T1-2	Single Threshold	0.3658	9.8196	0.0060	500
	Dual Threshold	0.4863	2.9652*	0.0960	500
	Triple Threshold	0.6497	2.4070*	0.0520	500
Robustness test model T1-3	Single Threshold	0.3658	9.2645***	0.0020	500
	Dual Threshold	0.6794	9.3612***	0.0080	500
	Triple Threshold	0.7295	7.3552**	0.0120	500
Internet-2 the threshold regulation model T2-1	Single Threshold	13.4349	4.1973**	0.0480	500
	Dual Threshold	14.6432	1.6447	0.1960	500
	Triple Threshold	14.7342	2.6021	0.1260	500
Endogenous test model T2-2	Single Threshold	14.1122	4.1048*	0.0580	500
	Dual Threshold	14.3846	6.8207**	0.0120	500
	Triple Threshold	14.6945	4.7479*	0.0520	500
Robustness test model T2-3	Single Threshold	12.2757	8.9587***	0.0040	500
	Dual Threshold	13.3915	3.8665*	0.0700	500
	Triple Threshold	14.5861	3.5095*	0.0540	500
Internet-3 the threshold regulation model T3-1	Single Threshold	7.1277	6.7898**	0.0180	500
	Dual Threshold	7.7235	6.6721***	0.0040	500
	Triple Threshold	7.9338	3.0765*	0.0820	500
Endogenous test model T3-2	Single Threshold	5.9572	5.5828**	0.0380	500
	Dual Threshold	6.7288	2.8531	0.1140	500
	Triple Threshold	7.1277	3.7413	0.1120	500
Robustness test model T-3-3	Single Threshold	7.7235	10.2633***	0.0020	500
	Dual Threshold	7.8215	10.4587***	0.0040	500
	Triple Threshold	8.0552	3.4298*	0.0620	500

^a^The estimation results are extracting the standard deviation of robustness

Secondly, the level of Internet penetration (Internet-2) was applied as a threshold regulating variable to test its iterative impact on economic spillovers of the sports industry (model T2-1 in Table 5). The results indicate that under the dynamic regulation of Internet penetration level, the economic spillover of the sports industry shows a "U" shape characteristic of first descending and then ascending when the Internet penetration rate lies in the first threshold range [0 13.4349], the development level of sports industry significantly inhibits the regional economic development; when the Internet penetration rate elevates to the second threshold range [13.4349 14.6432] and the third threshold range [14.6432 14.7342], the economic spillover impact of the sports industry is not significant, making it difficult to form an effective co-rail spillover; when the Internet penetration rate exceeds the turning point of 14.7342, the level of sports industry development significantly promotes regional economic development and leverages the economic spillover dividends of the sports industry. It is found through the above evolutionary law that a low-intensity level of Internet penetration has insignificant effects on the economic spillover drive of the sports industry and may even produce inhibitory effects. While a high-intensity level of Internet penetration will form a benign complementary mechanism with the sports industry, further releasing the economic growth power of the sports industry so as to drive the reconstruction and upgrading of the value chain of the sports industry.

Finally, by applying the level of Internet business application value (Internet-3) as a threshold regulation variable and testing its iterative spillover effect on regional economic development, it still remains the triple threshold (7.1277; 7.7235; 7.9338) law (model T3-1 in Table 5). The model demonstrates that when the level of Internet business application is below 7.1277, the elasticity coefficient of the impact of the level of sports industry development on regional economic developments is -1.6165, which passes the 1% significance level test and significantly inhibits the economic development of sports industry; when the level of Internet business application value reaches the second threshold range [7.1277 7.7235], the impact of the level of sports industry development on regional economic development impact becomes positive but insignificant; when the level of Internet business application value is advanced to the third threshold range [7.7235 7.9338], the impact of sports industry development level on regional economic development becomes negative and insignificant; when the level of Internet business application value is in the fourth threshold range [7.9338 +∞], the level of sports industry development releases a positive spillover effect on regional economic development. The general tendency reflects that the iterative impact of “Internet +” on the economic spillover of the sports industry is comparatively complex. However, in conjunction with the results of the linear model test, it is apparent that the sports industry has a positive and significant impact on regional economic development; hence, by strengthening the level of Internet business applications, the positive economic spillover of the sports industry can be driven in a particular degree, and new economic growth points of the sports industry can be shaped.

The results of the three Internet dimensional tests indicate that the economic spillover effects on the sports industry, whether it is the level of Internet infrastructure, Internet penetration, or Internet business application, generally present a “U” shaped pattern of first descending and then ascending. This common feature suggests that in the early stage of Internet development, the sports industry failed to effectively drive regional economic development, and there was a “crowding out effect”. The potential reasons for this are, for one thing, that the internet, as technological innovation, is a kind of “creative destruction” for the traditional sports industry. The regulations of the traditional sports elements market and its transactions have been broken and reshaped, and the value chain of the sports industry has been reconstructed, which has compelled the transformation of the sports industry to modernization without effectively promoting the regional economic development in the short term. For another, the impact of “Internet +” on the traditional sports industry is continuously expanding. The “virtual economy” generated by “Internet +” comprises the massive growth of Internet cultural and entertaining content, esports and Internet games occupy a substantial amount of young people’s spare time, which is not conducive to the healthy development of juveniles and compresses the scale of the consumer groups of the traditional sports industry and squeezes out the market space of the substantive economy, which may cause a “crowding out effect” on the development of the sports industry and inhibit the economic spillover of the sports industry. However, this inhibiting influence exists with a short break. When the level of Internet development exceeds a specific threshold, the scale of the information network, the improvement of information dissemination efficiency, and the reduction of information transaction costs will assist in achieving its market-oriented application. It will indirectly promote the sports industry to explore new market space, accelerate the scale of the target audience, propel the two-way reform transformation of the supply side and demand side of the sports industry, assist the sports industry in breaking the transformation bottleneck, expand the economic scale effect, and stimulate the economic spillover vitality of the sports industry.

### Endogenous control and robustness tests

#### Endogenous control

In order to reduce the endogenous disturbance of the core explanatory variables, firstly, the observations of the core explanatory variables and the explained variables were processed logarithmically to minimize the time trend disturbance of the macro data; secondly, the effects of the relevant control variables were examined in the econometric model. Meanwhile, the linear model was tested for fixed effects by incremental control variables. It is revealed that there is a positive relationship of dependence between the level of sports industry development and regional economic development, which further verifies the credibility of the linear estimation results (see model L2-5 in Table 4), therefore indicating that the linear estimation results reasonably control for endogenous effects.

We adopt the approach of Han Xianfeng et al. (2019) [52] in the dynamic threshold model, with the explanatory variables lagged by one order (models T1-2, T2-2, and T3-2 in Table 6). The results show that only moderate changes in thresholds and estimated coefficients emerged in the economic spillover path of the Internet development level to the sports industry. The compared results illustrate that the dynamic threshold estimation model outputs somewhat exclude endogenous disturbances.

**Table 6:** Test results of the iterative effect of the Internet on the economic spillover of the sports industry.

Variables	Internet-1 T1-1	Internet-1 T1-2	Internet-1 T1-3	Internet-2 T2-1	Internet-2 T2-2	Internet-2 T2-3	Internet-3 T3-1	Internet-3 T3-2	Internet-3 T3-3
$Internet_{0}^{1}-\alpha_{1}$	-0.9110** (-2.5093)	-0.5794*** (-3.0573)	-2.6440*** (-6.3598)	-1.1215** (-2.5902)	-0.7671*** (-3.4767)	-0.6133 (-1.1394)	-1.6165*** (-4.0946)	-0.4197** (-2.1189)	-1.6320*** (-4.3842)
$Iternet_{1}^{2}-\alpha_{2}$	0.0171 (0.5325)	-0.0308 (-1.4581)	-0.0856*** (-3.3885)	-0.0195 (-0.4911)	0.0023 (0.1589)	-0.1239*** (-4.0329)	0.0283 (0.7014)	0.0157 (0.7231)	-0.0661** (-2.0498)
$Internet_{2}^{3}-\alpha_{3}$	-0.0326 (-1.5106)	-0.0116 (-1.3706)	-0.0904*** (-3.8806)	-0.0380 (-1.2990)	-0.0046 (-0.4340)	-0.0666** (-2.6310)	-0.0038 (-0.1215)	-0.0124 (-0.9375)	-0.0845*** (-2.9988)
$Internet_{3}^{4}-\alpha_{4}$	1.0199*** (13.4488)	1.0510*** (28.4188)	1.3831*** (19.3318)	1.1933*** (13.1356)	0.9321*** (22.8251)	1.0858*** (12.6805)	1.1352*** (15.7061)	0.9653*** (21.4612)	1.1421*** (17.1512)

^a^$INT_{n}^{n+1}-\alpha_{n+1}$ denotes the difference in the elasticity coefficients of the impact of basic research on the level of technology when the level of Internet development is at different threshold ranges, and the results of the control variable estimation will not be repeated

#### Robustness tests

When testing the robustness of the linear estimation model, the regression method is altered to test whether the regression of macro data is subject to time trends in a random effect model (model L6 in Table 3). As the results show, the core explanatory variable of sports industry development level and the explanatory variable of regional economic development level maintain a positive correlation, similar to the output of the fixed-effects model. Only the elasticity coefficients and significance of some single control variables change to a certain extent, which verifies the robustness of the linear estimation results.

The dynamic threshold effect model lacks a replaceable test model, so we adjusted the data by removing the provinces and regions with the highest and lowest levels of economic development (models T1-3, T2-3, and T3-3 in Table 6) to reduce the extreme figure data interference. The results found that the output of the robustness model is fundamentally consistent with the dynamic basic model, thus confirming the credibility of the dynamic threshold model output.

### Regional heterogeneity analysis

The regional decomposition corresponding to the data statistics of the 11 provinces and regions in 2019 is presented in Table 7 below. In the Internet infrastructure dimension (INT-1), all six provinces and regions had already entered the positive innovation spillover level of Internet infrastructure, and only five provinces and regions, including Anhui, Hunan, etc., were in the negative spillover or insignificant level, requiring acceleration of new infrastructure to drive regional economic capacity release. In the Internet penetration dimension (INT-2), eight provinces and regions like Jiangxi and Anhui still had not yet attained the positive spillover level. It is worth focusing on that not all the provinces and regions with Internet penetration below the threshold level are western. However, most of them are central provinces with a large population, such as Anhui and Hunan, which could be explained by the large population base and the relatively low Internet penetration rate of the elderly and minors, slowing down the spillover effect of the “Internet” to a certain extent. In the Internet business application value dimension (INT-3), five provinces and regions, Hebei, Sichuan, Zhejiang, etc., stepped into the positive driving path of economic spillover from the sports industry. The remaining six provinces and regions were still at the negative spillover or insignificant level. This indicates that China’s Internet market’s value space is tremendous, but some regions’ Internet industry has not yet effectively occupied the market. Overall, China’s relatively high level of Internet development can effectively drive economic spillovers from the sports industry. However, there are certain uneven differences in the three dimensions. Firstly, among the three dimensions, the adjustment impact of Internet penetration on economic spillover from the sports industry only leverages spillover dividends in three provinces and regions, namely Zhejiang, Guangdong, and Sichuan; therefore, we should pay attention to the local improvement of Internet penetration. Second, the Internet industry is intensively distributed in the east and west, and the concentration of the Internet industry in the central region is relatively low, so the central provinces should enhance the Internet industry so as to better drive economic growth and achieve coordinated development among regions.

**Table 7:** Regional decomposition of the iterative impact of the level of Internet development on the economic spillover of the sports industry.

threshold regulation spillover effect	Internet infrastructure (INT-1)	Internet penetration (INT-2)	Internet business application Value (INT-3)
Positive spillover (α>0; p<0.1)	Eastern regions: Zhejiang, Guangdong, Shanghai West regions: Sichuan, Chongqing, Guizhou	Eastern regions: Zhejiang, Guangdong West regions: Sichuan	Eastern regions: Hebei, Zhejiang, Guangdong, Shandong, West regions: Sichuan
Critical threshold η	0.8790	14.7342	6.5898
Negative spillover(α<0; p<0.1) Or insignificant(p>0.1)	The other five provinces and regions	The other eight provinces and regions	The other six provinces and regions

## Discussion

In this study, we applied China’s inter-provincial panel data from 2015–2019 to examine the driving relationship between the sports industry development level and regional economic development and further explore the leverage effect of “Internet +” on the sports industry economic spillover. This study found a significant correlation between the sports industry and the regional economy, which is in consistent with previous studies [18, 50]. Similar to the result of Zhong [43] and Pan’s [34] study, that the integration of the sports industry and the Internet could boost China’s economic development, this study also verified a positive relationship of dependence between the impact of the sports industry on regional economic development and the regulation mechanism of the Internet. Compared to empirical research on the impact of the digital economy on sports development, which reveals that the digital economy is positive for the high-quality development of the sports industry at a significance level of 1% [49], this study signifies that the regulating effect of the Internet on the economic spillover of the sports industry has complex non-linear characteristics. Only when the level of Internet development exceeds a specific threshold can it develop a benign complementary mechanism with the sports industry, leveraging the economic spillover dividends of the sports industry and creating new economic growth points. This Internet threshold effect exists not only for the sports industry spillover but also for areas such as the manufacturing industry [57], and residential consumption [58]. Concerning the results of previous research, this study finds that regional heterogeneity exists in the sports industry’s impact on the regional economy [19] and the regulation effect of the Internet. The core problem for the regional heterogeneity is that each province’s Internet development level is difficult to coordinate in the three dimensions of Internet construction level, Internet penetration rate, and Internet business application value.

## Conclusion

This paper revealed a significant correlation between the sports industry and the regional economy. Furthermore, it verified a positive relationship of dependence between the impact of the sports industry on regional economic development and the regulation mechanism of the Internet. However, the regulating effect of the Internet on the economic spillover of the sports industry has complex non-linear characteristics. Only when the level of Internet development exceeds a specific threshold can it develop a benign complementary mechanism with the sports industry. Internet construction level, penetration rate, and business application value are three dimensions contributing to the regional heterogeneity of regulation effects.

The implication of the study findings for policies are as follows: Firstly, the sports industry’s development follows the market economy development principle. As a “sunrise industry”, the sports industry contains a massive commercial value, and with the development of the economy, the demand for the sports industry presents explosive growth, and the marginal consumption generated by the sports industry has released a vast market space. Simultaneously influenced by the industry linkage effect, the sports industry drives the rapid expansion of upstream and downstream industries and the industry linkage upgrade, leveraging economic development dividends. Secondly, the “Internet +” is the key force for transforming and upgrading the sports industry in the digital economy era. The Internet is used as a medium to break through the traditional business model of the sports industry, assist the reform of the supply side and demand side of the sports industry, and establish a new form of the sports industry based on the level of Internet development, drive the economic spillover of the sports industry, accelerate the new infrastructure, enhance the Internet penetration rate, expand Internet business applications, upgrade the level of Internet development to a specific point, release the drive of positive spillover, and realize the joint track evolution of “Internet +” and the sports industry. Thirdly, there is still regional heterogeneity among regions in the three dimensions of Internet infrastructure level, Internet penetration rate, and Internet business application value. In order to leverage the economic spillover effect of “Internet+” on the sports industry, in terms of Internet infrastructure construction, in the central region compared with the eastern and western regions, infrastructure scale coverage is relatively low, and more investment should be made to accelerate the new infrastructure and build the Internet technology platform. In terms of Internet penetration rate, the overall Internet penetration rate of each region requires advancement and should be accelerated to speed up the construction of the network and expand the audience scale. In terms of Internet business applications, the eastern region is significantly superior to the central and western regions. The eastern region should utilize the existing technological advantages to drive the transformation of the modern sports industry so as to better boost economic growth and achieve coordinated development between regions.

Theoretically and practically, this study provides some meaningful insights for future research work. From a theory perspective, there are questions that need to be further verified in future studies, for instance, whether the relationship between the sports industry and regional economy will be moderated by other confounding factors, whether the results will remain the same with statistics from other provinces and cities, and whether threshold regulation variables can fully reflect the level of the Internet development. From a practical perspective, more quantitative cohort research is needed to analyze the Internet’s influence and specific functional mechanism on the sports industry and regional economic development. Second, the transformation and upgrading of the sports industry through the digital economy have become a trend. As a result, more and more sports enterprises will invest more in Internet applications, digital sports security, and digital sports talents, and Meta plus sports will become an essential research direction in the future. Third, bringing prosperity to all is an essential requirement of socialism with Chinese characteristics, and well-balanced development is one of the premier goals of China’s development. Therefore, future research should focus on achieving a balanced development of the Internet in different regions in terms of Internet construction level, Internet penetration rate, and commercial Internet application, and thus promote the balanced development of the sports industry and economy and mitigate the heterogeneity in different regions.

## Supporting information

S1 Data All used data sets. It contains the data used in the article.
(XLSX)
