# Supplementary material for: An analysis of the leverage effect of “Internet +” on the economic spillover of sports industry
Source: PLoS One. 2023 Jul 19;18(7):e0288778. doi: 10.1371/journal.pone.0288778 (PMC10355464; doi:10.1371/journal.pone.0288778)
Supplement: Attachment — Submitted filename: Response to Reviewers.docx [file pone.0288778.s002.docx]

Dear Editor:

On behalf of all the contributing authors, I would like to express our sincere appreciation for your constructive comments concerning our article. These comments are all valuable and helpful for improving our article. According to your comments, we have extensively modified our manuscript and supplemented extra tables to make our results convincing. In the revised version, our manuscript’s changes were highlighted within the document using tracked changes in red. We offer a detailed point-by-point response to your insightful questions and suggestions below.

**Reviewer #1:**

**Q1.** The novelty of the paper is not validated by the authors. The paper should include a section in the introduction about the contribution of the paper as well as update the abstract.

**Response:** Thank you for this comment and suggestion; we do need to include the a section in the introduction about the contribution of the paper as well as update the abstract. Therefore, we rewrote the last paragraph of the introduction and updated the abstract.

Abstract

Given the unprecedented and profound impact on the traditional sports industry and its economic driving force brought by the “Internet +” , the article analyzes the driving relationship between the sports industry and local economic development and deconstructs the dynamic regulation mechanism of “Internet +” on the economic spillover of the sports industry. Empirical research based on inter-provincial panel data reveals that the leverage effect of economic spillover of the sports industry has significantly promoted regional economic growth, and “Internet +” functions as a two-way regulator. Only when the development level of the Internet exceeds a certain threshold can “Internet +” form an efficacious complementary mechanism with the sports industry. The economic spillover dividend of the sports industry will accelerate regional economic growth. However, the impact of “Internet +” on the threshold regulation of the sports industry economic spillover has spatial and temporal differentiation characteristics in the three dimensions of Internet hardware level, Internet penetration rate, and Internet application value. Therefore, it is required to apply differential regulation strategies in future policy design to realize the integration-driven and empower the high-quality development of “Internet+” and digital sports.

Introduction

The Internet has entered all circles of people’s lives, but its impact on the economic spillover effect of the sports industry is still uncertain. Therefore, how to stimulate the economic spillover effect of the sports industry in the context of the Internet is of great theoretical value and practical significance. This study concentrates on the economic spillover effect of the sports industry and utilizes China’s inter-provincial panel data from 2015-2019 to deconstruct the mechanism of the Internet. The purpose of this study was to: (1) explore the leverage effect of economic spillover of the sports industry in the context of “Internet +”; (2) examine what the specific regulation mechanism is; (3) investigate the practical needs of transformation of the sports industry and the dilemmas during the process of transformation. This study offers new insights into the role of the Internet in the process of the sports industry driving the regional economy and suggests future directions for sports industry research and government policies.

**Q2.** The related work does not support the problem identified in this study. There should be citations for the statistical figures reported in the paper. This literature review should include recent citations from 2020, 2021, and 2022.

**Response:** Thank you for this comment and examples, indeed, we also deeply believe that our manuscript should include more citations for the statistical figures from 2020, 2021, and 2022. Therefore, we added following citations in red in our manuscript.

(1) Line 117, page 6: In the study on the interaction between the sports industry and regional economic development, Zhang Liang noted that when the per capita disposable income (PCDI) of urban residents is less than 13659.26 yuan, increasing investment in the sports industry is not conducive to regional economic growth; in areas where the PCDI is between 13659.26 and 21168.79 yuan, the effect of investment in the sports industry on regional economic growth is not significant; when the PCDI exceeds 21168.79 yuan, increasing investment will significantly contribute to regional economic growth [28].
(2) Line 127, page 7: The added value of the sports industry is an important indicator that can reflect the development level of the sports industry. For example, from the data of 2017, the added value of the sports industry in Fujian, Guangdong, and Jiangsu in the eastern region is 132.401 billion yuan, 132.186 billion yuan, and 121.955 billion yuan, respectively. At the same time, the added value of the sports industry in Henan, Hunan, and Anhui in the central region reveals that the level of regional economic development not only directly affects the results but also determines the ability of their sports industry development [30].
(3) Line 133, page 7: The coupling degree between the sports industry and economic development in Shanghai in the eastern region was 0.4986, 0.4965, and 0.4992 from 2015 to 2017, respectively, and the coupling degree between the sports industry and economic development in other provinces during the same period was no higher than 0.5. The results reflect that the sports industry has been improving its quality and efficiency in recent years, with an average annual growth rate higher than that of GDP in the same period. However, the regional economy and sports industry system is a contradictory and unified; the two complement each other and promote each other to produce a combined effect, achieving sustainable and joint development [31].
(4) Line 153, page 8: By applying “internet+” to the sports industry, the innovative development of new formats such as Internet broadcasting, intelligent software and hardware, e-commerce, and esports can be realized. For instance, the revenue of sports software has increased from 108 million yuan in 2010 to 352 million yuan in 2021 [38].
(5) Line 170, page 8. The transformation of the sales of sports products in the context of “Internet +” has revolutionized the process of sports consumption, with material consumption such as sports equipment and apparel, and service consumption such as sports events and training changing to O2O (Online to Offline) mode, meanwhile, “Internet +” has also profoundly affected the consumption style and process of products including sports lottery, esports, sports copyright, physical education, and scientific research, and culture [42], for instance, according to the data of Taiwan Sports Lottery from May 1, 2018 to April 30, 2019, the betting rate with mobile phone and tablet computer was as high as 70% [43].
(6) Line 179, page 9. The shift of consumption characteristics in the context of “Internet +” has reconstructed the form of economic rents obtained by enterprises. In the age of Internet 2.0, the sharing model has become prevailing. The connecting dividend rental has gradually replaced the ability rental under the role of the Internet so that enterprises no longer make money by directly selling products, nor do they concentrate on chasing product sales dividends. Thus a great number of traditional fitness companies have already started to utilize their offline resources to enhance digital upgrades[44]. The shift was even more prominent after the outbreak. An analysis of 251 people revealed that 67.7% of respondents were not interested in visiting offline retail channels in the future [45].

**Q3.** The authors should consider the comparison of various existing studies in tabular form to validate the missing gap.

**Response:** Thank you for this comment, and we do believe that the tabular form can validate the missing gap. Therefore we added a form in the commentary part as follows:

Table 1: Different Research on Sports and Regional Economy

| **Region/Scope** | **Context** | **Research Methods** | **Research Subjects** |
| --- | --- | --- | --- |
| National | None | Quantitative | Core elements for coordinated development of regional economy and sports industry [22]. |
| 12 Provinces and Cities | None | Quantitative | The coupling degree and coordination degree of sports industry and regional economy [29]. |
| National | China’s New Era | Qualitative | The strategy of the sports industry contributes to the construction of economic power. The sports industry is conductive to the ecological economy of regional development [25]. |
| 12 Provinces and Cities | None | Quantitative | The relationship and heterogeneity of sports industry and regional economy [24]. |
| 12 Provinces and Cities | None | Quantitative | Regional characteristics of the sports industry development [28]. |
| 14 Provinces and Cities | Digital Economy | Quantitative | the Impact of Digital Economy on the High-Quality Development of Sports Industry.[49] |

**Q4.** The author should provide an analysis of the proposed study. Analyses can be based on the same methods as the above papers.

**Response:** Thank you for this comment, and we do reckon that adding an analysis of the proposed study would contribute more credibility to our study. Therefore, we added a similar study that applied the same data and similar methods in the commentary section. We rewrote the paragraph as follows:

As seen in Table 1, most Chinese scholars stated that the sports industry could assist in developing the regional economy. Some studies use the same database as this paper to analyze the correlation between the sports industry and regional economy and put forward the concepts of regional heterogeneity, coupling degree, and evaluation index, which provide rich research ideas and a theoretical basis for the study of the sports industry and regional economy. For instance, An analysis applied a time-fixed effects model to study the economic growth and sports industry structure in 12 provinces and cities in different regions of the country. Controlling for other variables, the total output and value-added of the sports industry were significant at the 1% level and contributed significantly to regional GDP. Most Chinese scholars stated that the sports industry could assist the development of the regional economy. Some of them analyzed the correlation between the sports industry and the regional economy, and researches on the impact of the sports industry on the regional economy is more abundant. The multi-dimensional investigation is combined with the industrial characteristics and geographical location, which provides a wealth of research ideas and theoretical foundations for the research on the sports industry and regional economy. However, it can be seen that there exist specific differences in the industrial development stage, industrial pattern, and market environment in China’s sports industry in different regions. Therefore, the results from a nationwide study can not fully represent the relationship between the sports industry and the regional economy.

In addition, in the background of industrial networking, domestic researchers have focused on the prospect and strategy of sports industry development under the context of “Internet +”, and a comprehensive analysis reveals that “Internet +” influences not only the sports business market but also the whole sports industry ecology, and the development of sports industry under the context of “Internet +” in China is still in the primary stage. The studies are mainly based on qualitative theoretical deduction research, which lacks objective quantitative research support, thus hindering the expansion of research profundity to a certain extent. This paper intends to analyze the driving relationship between the sports industry and regional economic development, explore the profound and intrinsic attributes of the sports industry in the context of “Internet +”, and deconstruct the dynamic regulation mechanism of Internet +” on the economic spillover of the sports industry by applying statistics and econometric model, so as to promote the integration and drive of “Internet +” and digital sports, and empower high-quality development in China.

**Q5.** Using benchmarks defined in relevant research, the author should consider a one-to-one comparison of the existing model and the proposed model.

**Response**: Thank you for your comments and suggestion, and we believed it’s more convincing to present a comparison of the existing model and the proposed model. Therefore, we corrected the econometric model part and list the advantages of proposed model as follows:

In this paper, we applied Hanse’s panel threshold regression model. Previous research utilized group testing or constructed multiplicative estimation models to analyze nonlinear threshold characteristics. However, both methods have certain shortcomings. First, it is difficult to solidify the grouping criteria in group testing, which may distort threshold estimation and lack of corresponding significance test. The multiplicative estimation model can estimate the overall impact threshold, but the cross-term cannot effectively analyze the impact relationship between core explanatory variables and threshold variables. The estimation results have limitations in interpretation. Hansen’s panel threshold regression model effectively compensates for the technical shortcomings of the above two threshold tests by constructing piecewise functions of the regression coefficients of the explanatory variables, which can not only estimate the thresholds but also further test the reasonableness of the thresholds for significance.

**Q6.** The author should identify future research directions.

**Response:** Thank you for your careful reading and suggestion, and we proposed future research directions in the conclusion part as follows:

Theoretically and practically, this study provides some meaningful insights for future research work. From a theory perspective, there are questions that need to be further verified in future studies, for instance, whether the relationship between the sports industry and regional economy will be moderated by other confounding factors, whether the results will remain the same with statistics from other provinces and cities, and whether threshold regulation variables can fully reflect the level of the Internet development. From a practical perspective, more quantitative cohort research is needed to analyze the Internet's influence and specific functional mechanism on the sports industry and regional economic development. Second, the transformation and upgrading of the sports industry through the digital economy have become a trend. As a result, more and more sports enterprises will invest more in Internet applications, digital sports security, and digital sports talents, and Meta plus sports will become an essential research direction in the future. Third, bringing prosperity to all is an essential requirement of socialism with Chinese characteristics, and well-balanced development is one of the premier goals of China’s development. Therefore, future research should focus on achieving a balanced development of the Internet in different regions in terms of Internet construction level, Internet penetration rate, and commercial Internet application, and thus promote the balanced development of the sports industry and economy and mitigate the heterogeneity in different regions.

**Reviewer #2:**

**Q1.** Keywords: Please add keywords.

**Response**: Thank you for your careful reading and rigorous attitude, and we are really sorry for our careless mistakes. We have added the keywords in the manuscript as follows:

**Keywords**: sports industry, “Internet +”, economic spillover, leverage effect, digital sports

**Q2.** Introduction: Please add references in the first few lines of the 1st paragraph.

**Response**: Thank you for this comment, and we believe adding references in the first few lines of the 1st paragraph is more convincing. Thus, we added the following references.

Entering the new era, the role of China's sports industry in the national economic and social development has become increasingly prominent [1], which has become a new growth point of the national economy, and the sports industry plays a highly significant role in facilitating the construction of an economic power, the construction of Healthy China and the construction of a sports power [2]. At the third session of the 12th National People’s Congress, the “Internet +” action program was first proposed by Premier Li Keqiang in the government work report [3].

[1] Ren B, Huang H. [Practical Problems and Development Strategies of China's Sports Industry Under the Background of Sporting Powerhouse Construction](https://oversea.cnki.net/kns/Detail?sfield=fn&QueryID=0&CurRec=1&recid=&FileName=TYWS202204011&DbName=CJFDLAST2022&DbCode=CJFD&yx=&pr=&URLID=). [Sports Culture Guide](https://oversea.cnki.net/kns/Navi?DBCode=CJFD&BaseID=TYWS). 2022; (04):68-74+89. doi:CNKI:SUN:TYWS.0.2022-04-011.

[2] Huang H, Kang L. [heoretical Logic and Implementation Path of High-Quality Development of Sports Industry in the New Era](https://oversea.cnki.net/kns/Detail?sfield=fn&QueryID=0&CurRec=1&recid=&FileName=TYKX202201002&DbName=CJFDLAST2022&DbCode=CJFD&yx=&pr=&URLID=).China Sport Science. 2022; 42(01):15-34+58.DOI:10.16469/j.css.202201002.

[3] Li K. REPORT ON THE WORK OF THE GOVERNMENT-Delivered at the Third Session of the 12th National People’s Congress of the People’s Republic of China on March 5, 2015. Available from: http://www.gov.cn/guowuyuan/2015-03/16/content_2835101.htm (accessed 24th January 2023)

**Q3.** Introduction: Please clearly mention the uniqueness of your study in the first paragraph.

**Response**: Thank you for your careful reading, and we do believe that it will be more clear and understandable to add the uniqueness in the first paragraph. Thus, we added the following in the first paragraph of the introduction part.

The downward pressure on China’s economic growth is rising continuously, and the increasing trend of the sports industry can not only hedge the risk to some extent but also contribute to industrial restructuring. Therefore, this study concentrates on analyzing the relationship between the sports industry and regional economic development and how to maximize the spillover effect of the sports industry to promote the regional economy’s development better.

Q4. Introduction: The 1st paragraph is extra large I suggest to split into two paragraphs and make it logical.

**Response**: Thank you for your careful reading, and we rewrote the first paragraph and split it into two paragraphs logically as follows:

Entering the new era, the role of China's sports industry in the national economic and social development has become increasingly prominent [1], which has become a new growth point of the national economy, and the sports industry plays a highly significant role in facilitating the construction of an economic power, the construction of Healthy China and the construction of a sports power [2]. At the third session of the 12th National People’s Congress, the “Internet +” action program was first proposed by Premier Li Keqiang in the government work report [3]. The sports industry in China maintained a torrid growth during 2016-2019, as the total scale increased beyond a trillion yuan, up by 55.1%, which is 1.14% of GDP surged from 0.87%.

Due to the Covid-19 pandemic, the speed of economic growth of the national value-added of the tertiary industry slowed sharply in 2020 [4]. While the sports industry transformed to digital sports with the help of new technologies, such as big data[5], “Internet +” [6], 5G [7], etc., the value-added of sports media and information service industry maintained 18.9% growth, and the value-added of physical education and training industry expanded 5.7% through new business models like livestreaming fitness[8] and online training [9]. The sports industry’s economic effect in China is getting gradually prominent. The downward pressure on China's economic growth is rising continuously, and the increasing trend of the sports industry can not only hedge the risk to some extent but also contribute to industrial restructuring. Therefore, this study concentrates on analyzing the relationship between the sports industry and regional economic development and how to maximize the spillover effect of the sports industry to promote the regional economy's development better.

Q5. Discussion: Compare your result with previous similar studies

**Response**: Thank you for your comments, and we all consider it necessary to list comparisons. Therefore, we rewrote the first paragraph of the discussion part as follows:

In this study, we applied China’s inter-provincial panel data from 2015-2019 to examine the driving relationship between the sports industry development level and regional economic development and further explore the economic spillover impact of "Internet +" on the sports industry. Similar to a previous study on the relationship and heterogeneity between the sports industry and the regional economy [26], this study found a significant correlation between the sports industry and the regional economy. However, this study also verified a positive relationship of dependence between the impact of the sports industry on regional economic growth and the regulation mechanism of the Internet. Compared to empirical research on the impact of the digital economy on sports development which reveals that the digital economy is positive for the high-quality development of the sports industry at a significance level of 1% [49], this study signifies that the regulating effect of the Internet on the economic spillover of the sports industry has complex non-linear characteristics. Only when the level of Internet development exceeds a specific threshold can it develop a benign complementary mechanism with the sports industry, leveraging the economic spillover dividends of the sports industry and creating new economic growth points. As the previous study reveals, regional heterogeneity exists in the sports industry’s impact on the regional economy. This study also found regional heterogeneity in the regulation effect of the Internet. The core problem is that each province’s Internet development level is difficult to coordinate in the three dimensions of Internet construction level, Internet penetration rate, and Internet business application value.

Q6. Please mention future work in the conclusion.

**Response:** Thank you for your comment and suggestion, and we highly agree that it is necessary to propose future research directions in the conclusion part. Therefore, we added future research directions in the conclusion part as follows:

Theoretically and practically, this study provides some meaningful insights for future research work. From a theory perspective, there are questions that need to be further verified in future studies, for instance, whether the relationship between the sports industry and regional economy will be moderated by other confounding factors, whether the results will remain the same with statistics from other provinces and cities, and whether threshold regulation variables can fully reflect the level of the Internet development. From a practical perspective, more quantitative cohort research is needed to analyze the Internet's influence and specific functional mechanism on the sports industry and regional economic development. Second, the transformation and upgrading of the sports industry through the digital economy have become a trend. As a result, more and more sports enterprises will invest more in Internet applications, digital sports security, and digital sports talents, and Meta plus sports will become an essential research direction in the future. Third, bringing prosperity to all is an essential requirement of socialism with Chinese characteristics, and well-balanced development is one of the premier goals of China’s development. Therefore, future research should focus on achieving a balanced development of the Internet in different regions in terms of Internet construction level, Internet penetration rate, and commercial Internet application, and thus promote the balanced development of the sports industry and economy and mitigate the heterogeneity in different regions.

Q7. Please add the conclusion part.

**Response:** Thank you for your comment and suggestion, and we have added the the conclusion part in the revised manuscript as follows:

**Conclusion**

This paper revealed a significant correlation between the sports industry and the regional economy. Furthermore, it verified a positive relationship of dependence between the impact of the sports industry on regional economic growth and the regulation mechanism of the Internet. However, the regulating effect of the Internet on the economic spillover of the sports industry has complex non-linear characteristics. Only when the level of Internet development exceeds a specific threshold can it develop a benign complementary mechanism with the sports industry. Internet construction level, penetration rate, and business application value are three dimensions contributing to the regional heterogeneity of regulation effects. According to the results, this study also promotes suggestions for future policies. The sports industry’s development follows the market economy development principle, and the generated marginal consumption has already expanded market space. Thus, it is a practical choice for local governments to develop the economy through the sports industry. Moreover, the “Internet +” is the binding force for transforming and upgrading the sports industry in the digital economy era. Therefore, it is necessary for provinces and cities to enhance the Internet penetration rate, expand Internet business applications, and upgrade the level of Internet development to a specific point to maximize the drive of positive spillover. The regional heterogeneity nature requires more concentration on the middle and western regions.

Theoretically and practically, this study provides some meaningful insights for future research work. From a theory perspective, there are questions that need to be further verified in future studies, for instance, whether the relationship between the sports industry and regional economy will be moderated by other confounding factors, whether the results will remain the same with statistics from other provinces and cities, and whether threshold regulation variables can fully reflect the level of the Internet development. From a practical perspective, more quantitative cohort research is needed to analyze the Internet's influence and specific functional mechanism on the sports industry and regional economic development. Second, the transformation and upgrading of the sports industry through the digital economy have become a trend. As a result, more and more sports enterprises will invest more in Internet applications, digital sports security, and digital sports talents, and Meta plus sports will become an essential research direction in the future. Third, bringing prosperity to all is an essential requirement of socialism with Chinese characteristics, and well-balanced development is one of the premier goals of China’s development. Therefore, future research should focus on achieving a balanced development of the Internet in different regions in terms of Internet construction level, Internet penetration rate, and commercial Internet application, and thus promote the balanced development of the sports industry and economy and mitigate the heterogeneity in different regions.
