# Supplementary material for: An analysis of the leverage effect of “Internet +” on the economic spillover of sports industry
Source: PLoS One. 2023 Jul 19;18(7):e0288778. doi: 10.1371/journal.pone.0288778 (PMC10355464; doi:10.1371/journal.pone.0288778)
Supplement: Attachment — Submitted filename: review-plos.docx [file pone.0288778.s003.docx]

Thank you for considering given comments seriously and providing response comprehensively.

However this may add an ice to the cake if you make following changes/additions.

- Add a small paragraph at the end of introduction section and provide key findings.
- Also add the structure of paper soon after providing key findings.
- Adjust table 05 properly

Good luck.
