# Supplementary material for: An analysis of the leverage effect of “Internet +” on the economic spillover of sports industry
Source: PLoS One. 2023 Jul 19;18(7):e0288778. doi: 10.1371/journal.pone.0288778 (PMC10355464; doi:10.1371/journal.pone.0288778)
Supplement: Attachment — Submitted filename: Response to Reviewers.docx [file pone.0288778.s004.docx]

Dear Editor and reviewers:

On behalf of all the contributing authors, I would like to express our gratitude for your helpful feedback on our article. Your comments have been invaluable in improving our work. According to your suggestions, we have extensively modified our manuscript and added extra tables to make our results convincing. We have highlighted all the changes made to the manuscript using tracked changes in red. We offer a detailed point-by-point response to your insightful questions and suggestions below.

Editor:

**Q1** Please review your reference list to ensure that it is complete and correct.

**Response:** Thank you for your valuable suggestion. To ensure the reference list is complete and correct, we reviewed all references in the manuscript. Firstly, we checked all references to see whether the article was retracted. Secondly, to make the article more rigorous and convincing, we added eight new references in the revised manuscript compared with the original one. Furthermore, we re-edited the reference format of the article by Endnote to make it consistent with the journal’s requirements. The reference order of the revised manuscript has changed compared to the original manuscript due to the new paragraphs we have added to the article. We have marked the changes in red in the manuscript with track changes and listed the comparison of the reference order of the two manuscripts in the table below.

| Number | |
| --- | --- |
| In the Original Manuscript | In the Revised Manuscript |
| [1]-[16] | [1]-[16] |
| [17]-[25] | [20]-[28] |
| [26] | [19] |
| [27] | [18] |
| [28]-[48] | [29]-[49] |
| [49] | [17] |
| [50] | [52] |
| - | [50],[51],[53],[54],[55],[56],[57],[58] |

**Q2** Please ensure that you refer to Table 2 in your text as, if accepted, production will need this reference to link the reader to the Table.

**Response:** Thank you for your careful reading and valuable suggestion. We are really sorry for this careless mistake. Therefore, we cited Table 2 in the paragraphs of Explained variables, Explanatory variables, Threshold regulation variables, and Control variables, respectively, and the revised section of Measurement of variables are as follows:

Explained variables: GDP, as an important evaluation index of macroeconomic output, can reflect the fundamentals of economic development in different regions, and the GDP growth rate is chosen to reflect the economic development level of different regions in the model regression, as shown in Table 2.

Explanatory variables: The sports industry is an essential part of national economic development. To assess the level of the sports industry development (Sport), we take the scale of regional sports industry output value as the evaluation criterion and do logarithmic treatment in the regression to reduce the influence of variance, as displayed in Table 2.

Threshold regulation variables: the level of “Internet +” development (Internet). In order to comprehensively reflect the development level of “Internet +”, we referred to Han’s [52] research method and decomposed the Internet development level into Internet construction level (Internet-1), which is measured by the distance of Internet lines laid in each province and region; Internet penetration rate (Internet-2), which is reflected by the total number of Internet users in each region; and Internet commercial application value (Internet-3), which is expressed by the commercial transaction amount of regional Internet data flow (Table 2).

Control variables: The selection of control variables should be based on theoretical foundations and previous studies, and those variables that may interfere with the correlation between explanatory and explained variables should be selected. According to the current literature, urbanization [53], trade [54], human capital [55], and capital stock [56] may affect the correlation between the explanatory and the explained variables. Therefore, to achieve unbiased test results and consider the measurement and operationalization of the variables, this study includes relevant control variables affecting economic development in the model. Specifically, these include: Urbanization, measured by the proportion of the urban population in the region; humans, reflected by the number of years of education per capita in the region; Trade, measured by the ratio of import and export volume to GDP in the region; and Capital is evaluated by the region’s capital stock in the current year and treated as a logarithm in the regression (Table 2).

Reviewer #3:

**Q1&Q2** Add a small paragraph at the end of introduction section and provide key findings. Also add the structure of paper soon after providing key findings.

**Response:** Thank you for your valuable suggestion. We believe it would be clearer if we added the structure and key findings of the paper at the end of the introduction section. Therefore, we added the following paragraphs.

A linear estimation model with sports industry economic spillover as the dependent variable was designed based on the previous studies. The level of “Internet +” development was employed as the threshold regulation variable to examine its dynamic impact on the economic spillover of the sports industry. Inter-provincial panel data from 11 provinces and regions from 2015-2019 were collected and analyzed through the fixed-effects model, threshold model, robustness test, and heterogeneity analysis. This study enhances the current research by analyzing the sports economic spillover on the regional economy under the influence of the Internet. It supports the findings of Ruan’s study [17] and extends the theoretical study of Huang et al [18] by incorporating the Internet to explain the sports spillover effects on the regional economy through empirical analysis. Moreover, this study also validates the results of Jiang’s research [19] on the sports industry and regional economy heterogeneity.

The study finds a significant correlation between the sports industry and the regional economy. A positive relationship of dependence between the impact of the sports industry on regional economic development and the regulation mechanism of the Internet is significant, but the regulating effect of the Internet on the economic spillover of the sports industry has complex non-linear characteristics. Only when the level of Internet development exceeds a specific threshold can it develop a benign complementary mechanism with the sports industry. Based on the empirical results, this study suggests that it is a practical choice for local governments to develop the economy through the sports industry. As the “Internet +” is the binding force for transforming and upgrading the sports industry, provinces, and cities must enhance the Internet penetration rate, expand Internet business applications, and upgrade the level of Internet development to a specific point to maximize the drive of positive spillover. The regional heterogeneity nature requires more concentration on the middle and western regions.

**Q3** Adjust table 05 properly

**Response:** Thank you for your suggestions. We are very sorry that we did not adjust Table 5 to meet the format required by the journal. Therefore, we have made proper adjustments to Table 6 in the revised manuscript (Table 5 in the original manuscript).

Reviewer #4:

**Q1** I noticed that the title suggests that “economic spillover” is the dependent variable (DV), as indicated by the keywords. However, the authors switch between the terms “economic development” and “economic growth”, which have distinct meanings and measures and are not necessarily proxies for economic spillover. Therefore, I would recommend that the authors clarify and be consistent about what they are analyzing as the DV, and ensure that it is clearly stated in the title, keywords, and throughout the text.

**Response:** Thank you for your suggestions. We do also believe that “economic spillover” is different from “economic development” and “economic growth”, and they can not simply replace each other. We are very sorry for the confusion caused by the choice of terms. We employed “economic spillover” to present the effect of the sports industry on the regional economy, and we also examined whether the Internet regulated this direct spillover effect. Therefore, to be more consistent and rigorous, we have standardized the terminology by integrating the terms “economic growth” and “economic development” and including the description of the spillover effect.

**Q2** Secondly, I would suggest that the authors consider adding a brief paragraph or two in the introduction section to summarize the policy implications and key results of their study. This would provide readers with a clear understanding of the potential real-world applications of the research and help to contextualize the findings.

**Response:** Thank you for your precious suggestion. We also consider that it will be more friendly for readers, if we could summarize their study’s policy implications and key results in the introduction section. Therefore, we added the following paragraphs.

A linear estimation model with sports industry economic spillover as the dependent variable was designed based on the previous studies. The level of “Internet +” development was employed as the threshold regulation variable to examine its dynamic impact on the economic spillover of the sports industry. Inter-provincial panel data from 11 provinces and regions from 2015-2019 were collected and analyzed through the fixed-effects model, threshold model, robustness test, and heterogeneity analysis. This study enhances the current research by analyzing the sports economic spillover on the regional economy under the influence of the Internet. It supports the findings of Ruan’s study [17] and extends the theoretical study of Huang et al [18] by incorporating the Internet to explain the sports spillover effects on the regional economy through empirical analysis. Moreover, this study also validates the results of Jiang’s research [19] on the sports industry and regional economy heterogeneity.

The study finds a significant correlation between the sports industry and the regional economy. A positive relationship of dependence between the impact of the sports industry on regional economic development and the regulation mechanism of the Internet is significant, but the regulating effect of the Internet on the economic spillover of the sports industry has complex non-linear characteristics. Only when the level of Internet development exceeds a specific threshold can it develop a benign complementary mechanism with the sports industry. Based on the empirical results, this study suggests that it is a practical choice for local governments to develop the economy through the sports industry. As the “Internet +” is the binding force for transforming and upgrading the sports industry, provinces, and cities must enhance the Internet penetration rate, expand Internet business applications, and upgrade the level of Internet development to a specific point to maximize the drive of positive spillover. The regional heterogeneity nature requires more concentration on the middle and western regions.

Reviewer #5:

**Abstract**

**Q1** The Abstract is the “Face” of the study, it must be high quality and informative. The abstract should be very specific to the aims, methodology, findings, and implications. Please focus the abstract on your study objectives and results. Results should be supported by their significance. Study implications should be discussed and based on the results.

**Response:** Thank you for your suggestions. We are all highly agree that the abstract should be more concise and specific to the objectives and results. Therefore, we concentrated more on the objectives and results and rearranged the abstract in a structural framework as follows:

Given the unprecedented and profound impact on the traditional sports industry and its economic driving force brought by the “Internet +”, the purpose of this study is to explore the driving relationship between the sports industry and local economic development and deconstruct the dynamic regulation mechanism of “Internet +” on the economic spillover of the sports industry. Empirical data collected from China’s 11 inter-provincial panel data from 2015-2019 were analyzed through the fixed-effects and threshold models. The study finds that the economic spillover of the sports industry has significantly promoted regional economic development, and the regulation effect of the Internet has complex non-linear characteristics. Only when Internet development exceeds a specific threshold can it develop a benign complementary mechanism with the sports industry. The impact of “Internet +” on the threshold regulation of the sports industry economic spillover has spatial and temporal differentiation characteristics in the three dimensions of Internet hardware level, Internet penetration rate, and Internet application value. The results of this study extend the current study, indicating that it is a practical choice for local governments to develop the economy through the sports industry. Provinces and cities should enhance the Internet penetration rate, expand Internet business applications, and upgrade Internet development to a specific point to maximize the drive of positive spillover. Regional heterogeneity requires differential regulation strategies and more concentration on the middle and western regions.

**Introduction**

**Q2** The paper lacks arguments on how and in what aspects it contributes to the literature. Objectives of the paper needs to be linked to the literature to identify how this paper is different than the previous ones and how it contributes to the literature on leverage effect of “Internet +” on the economic spillover of sports industry.

**Response:** Thank you for your valuable suggestion. Therefore, we added a description of the comparison of the results of this paper with other literature in the last part of the introduction, which also presents the additional contribution of this study to the literature. The added sections are as follows:

In page 4, line 104: A linear estimation model with sports industry economic spillover as the dependent variable was designed based on the previous studies. The level of “Internet +” development was employed as the threshold regulation variable to examine its dynamic impact on the economic spillover of the sports industry. Inter-provincial panel data from 11 provinces and regions from 2015-2019 were collected and analyzed through the fixed-effects model, threshold model, robustness test, and heterogeneity analysis. This study enhances the current research by analyzing the sports economic spillover on the regional economy under the influence of the Internet. It supports the findings of Ruan’s study [17] and extends the theoretical study of Huang et al [18] by incorporating the Internet to explain the sports spillover effects on the regional economy through empirical analysis. Moreover, this study also validates the results of Jiang’s research [19] on the sports industry and regional economy heterogeneity.

**Q3** Justification of the topic is too weak, and not supported by the strong empirical work.

**Response:** Thank you for your comment. We also believe aspects of the empirical argumentation for this theme could still be strengthened. Since the domestic literature directly related to this study is scarce, especially the empirical quantitative studies, the argumentation on the topic may not be sufficiently demonstrated. We hope that the results of this study derived from the existing literature and data will support future in-depth studies.

**Q4** The author did not mention clearly about his/her contribution of the study, which should be included at the end of the introduction.

**Response:** Thank you for your valuable suggestion, and we also think including the study’s contribution is necessary. Therefore, we add the following paragraphs at the end of the introduction section.

In page 5, line 109: This study enhances the current research by analyzing the sports economic spillover on the regional economy under the influence of the Internet. It supports the findings of Ruan’s study [17] and extends the theoretical study of Huang et al [18] by incorporating the Internet to explain the sports spillover effects on the regional economy through empirical analysis. Moreover, this study also validates the results of Jiang’s research [19] on the sports industry and regional economy heterogeneity.

The study finds a significant correlation between the sports industry and the regional economy. A positive relationship of dependence between the impact of the sports industry on regional economic development and the regulation mechanism of the Internet is significant, but the regulating effect of the Internet on the economic spillover of the sports industry has complex non-linear characteristics. Only when the level of Internet development exceeds a specific threshold can it develop a benign complementary mechanism with the sports industry. Based on the empirical results, this study suggests that it is a practical choice for local governments to develop the economy through the sports industry. As the “Internet +” is the binding force for transforming and upgrading the sports industry, provinces, and cities must enhance the Internet penetration rate, expand Internet business applications, and upgrade the level of Internet development to a specific point to maximize the drive of positive spillover. The regional heterogeneity nature requires more concentration on the middle and western regions.

**Literature review**

**Q5** A good literature review is included in the paper, but still no arguments can be seen in the selection of the study variables, theorization part in the literature review is weak.

**Response:** Thank you for your suggestion and comments. We have conducted some discussions on the selection of variables in the material and method section, and we also believe that it is essential to add a discussion on the selection of variables in the literature review section. Therefore, we added the following literature and statements in the literature review and material and method section, respectively, to enhance the theoretical part.

In page 11, line 260: For instance, a time-fixed effects model with the scale and value-added of regional sports industry output as the independent variable was applied to examine the economic growth and sports industry structure in 12 provinces and cities in different country regions. Controlling for other variables, the total output and value-added of the sports industry are significant at the 1% level and contribute significantly to regional GDP.

In page 12, line 264: Besides, Sun investigated the mechanism of sports consumption boosting urban economic development through panel data of major cities in Jiangsu Province, using the level of sports consumption as the core explanatory variable.

In page 12, line 277: For example, Ruan’s study employed a random effects model with the level of development of the digital economy as the explanatory variable to investigate its effect on the structure of the sports industry.

In page 15, line 337: Therefore, in the following section, we designed an econometric model with the sports industry development as the explanatory variable and the Internet development as the regulation variable to test the dynamic impact of “Internet +” on the economic spillover of the sports industry through empirical research.

**Q6** More contextual review should be included.

**Response:** Thank you for your suggestion. Indeed, it would be more convincing, if we could add diverse contextual review in the study. Thus, we added literatures of different contexts to enrich our study, and listed them in Table 1.

**Methodology**

**Q7** Why author has selected time period from 2015-2019? Provide rational for it.

**Response:** Thank you for your recommendation. We all believe that it will be more convincing if we could provide the reason for selecting the research period. Therefore, we add the following in the Data sources and testing section.

In page 19, line 436: In 2014, the General Office of the State Council issued the Opinions on Promoting National Fitness, Sports Consumption, and High-Quality Development of the Sports Industry. Since then, China’s sports industry’s added value and growth rate have grown significantly. However, the rapid development of the sports industry was hampered by Coivd-19 pandemic in 2020. Therefore, this paper selects the research period from 2015-2019, which is more representative.

**Q8** Hanse’s panel threshold regression model is used for the study analysis, no argumentation/healthy discussion is provided about the suitability of this regression model.

**Response:** Thank you for this comment, and we also deeply believe that it is necessary to include the discussion of selecting the regression model. Since we have promoted the advantage of Hanse’s panel threshold regression model compared to group testing or constructed multiplicative estimation models, we added the following to explain the suitability comprehensively.

In page 16 line 374: The model captures this nonlinear relationship when there is a threshold effect, i.e., when the relationship between the explanatory and explained variables changes significantly at a threshold point. The data utilized in the study are rather balanced and stable, and the sample size is relatively large, while this contributes to a more accurate estimation of the threshold and related parameters. Also, one of the purposes of this study was to investigate the threshold regulation effect of the Internet on the economic spillover of the sports industry. Therefore, this model fits the purpose and data requirements of this study.

**Q9** Operationalization of the variables may be provided in tabular form.

**Response:** Thank you for this comment, and we do believe that it is necessary to provide the tabular form to present the operationalization of variables. Therefore, we add the following table (Table 2 in the revised manuscript) in the section of the measurement of variables.

| Variables | Operation Definition | Indicator | Measure |
| --- | --- | --- | --- |
| GDP | the economic development level of the regions | GDP growth rate | Ratio |
| Sport | the level of sports industry development | the scale of regional sports industry output value | Ratio |
| Internet-1 | Internet construction level | the distance of Internet lines laid in each province and region | Ratio |
| Internet-2 | Internet penetration rate | the total number of Internet users in each region | Ratio |
| Internet-3 | Internet commercial application value | commercial transaction amount of regional Internet data flow | Ratio |
| Urbanization | the level of urbanization | the proportion of the urban population | Ratio |
| Human | human resources conditions | the number of years of education per capital in the region | Ratio |
| Trade | the contribution of the trade | the ratio of import and export volume to GDP in the region | Ratio |
| Capital | the total productive capital owned by an economy | region’s capital stock in the current year | Ratio |

**Results**

**Q10** The author must provide the rationale for choosing the control variables such as Urbanization and trade.

**Response:** Thank you for your suggestion, and we also think that it will be more convincing if we could add the explanation or choosing the control variables. Therefore, we rewrote the paragraph in the control variable part of measurement of variables and added four literatures to support the selection.

In page18, line 414: The selection of control variables should be based on theoretical foundations and previous studies, and those variables that may interfere with the correlation between explanatory and explained variables should be selected. According to the current literature, urbanization [53], trade [54], human capital [55], and capital stock [56] may affect the correlation between the explanatory and the explained variables. Therefore, to achieve unbiased test results and consider the measurement and operationalization of the variables, this study includes relevant control variables affecting economic development in the model. Specifically, these include: Urbanization, measured by the proportion of the urban population in the region; humans, reflected by the number of years of education per capita in the region; Trade, measured by the ratio of import and export volume to GDP in the region; and Capital is evaluated by the region’s capital stock in the current year and treated as a logarithm in the regression.

**Q11** The assumptions of Hanse’s model are not tested.

**Response:** Thank you for your comment. We also believe that it is necessary to test the model, thus, we applied endogenous control and robustness tests in the study. In the linear estimation model, to reduce the endogenous disturbance, the study logarithmically processed the observations variables and tested the linear model through fixed effects by incremental control variables. Meanwhile, the study altered the regression method to test the robustness of the original model through a random effects model. In the dynamic threshold estimation model, this study lagged the explanatory variables by one order to exclude endogenous disturbances. At the same time, we adjusted the data by removing the extreme figure interference to confirm the credibility. Also, before selecting the model, the study employed the Hausman test, which needed to be specifically described. Therefore, to be more rigorous, the following were added to the first part of the result section:

In page 21, line 468: Three models are usually available for panel data analysis: the mixed estimation model, fixed effects model, and random effects model. However, the differences in time and cross-sectional dimensions of the data in this paper are relatively slight, and the p-value of the Hausman test is not significant, i.e., an individual fixed effect exists. Therefore, this study applied the fixed-effects model to assess the linear relationship between the level of sports industry development and regional economic development.

**Q12** Results of the model are only discussed statistically but not supported by the previous literature.

**Response:** Thank you for your comment. We do agree that it would be more persuasive to discuss the results by combining statistical data and literature. Therefore, we rewrote the first paragraph of the discussion part as follows:

In page 33, line 696: In this study, we applied China’s inter-provincial panel data from 2015-2019 to examine the driving relationship between the sports industry development level and regional economic development and further explore the leverage effect of “Internet +” on the sports industry economic spillover. This study found a significant correlation between the sports industry and the regional economy, which is in consistent with previous studies [18, 50]. Similar to the result of Zhong [43] and Pan’s [34] study, that the integration of the sports industry and the Internet could boost China’s economic development, this study also verified a positive relationship of dependence between the impact of the sports industry on regional economic development and the regulation mechanism of the Internet. Compared to empirical research on the impact of the digital economy on sports development which reveals that the digital economy is positive for the high-quality development of the sports industry at a significance level of 1% [49], this study signifies that the regulating effect of the Internet on the economic spillover of the sports industry has complex non-linear characteristics. Only when the level of Internet development exceeds a specific threshold can it develop a benign complementary mechanism with the sports industry, leveraging the economic spillover dividends of the sports industry and creating new economic growth points. This Internet threshold effect exists not only for the sports industry spillover, but also for areas such as manufacturing industry [57], residential consumption [58]. Concerning the results of previous research, this study finds that regional heterogeneity exists in the sports industry’s impact on the regional economy [19] and the regulation effect of the Internet. The core problem for the regional heterogeneity is that each province’s Internet development level is difficult to coordinate in the three dimensions of Internet construction level, Internet penetration rate, and Internet business application value.

**Q13** The discussion is not based on latest literature. Support your discussion with recent literature.

**Response:** Thank you for your comment. We also consider that recent literature should be added to the discussion section to improve the temporal validity of the study and to endorse the findings. Therefore, based on the original literature, we added some latest literature to the discussion section and listed them in the table as follows:

| No. | Year | Title |
| --- | --- | --- |
| 49 | 2021 | Problems in the Development of China's Sports Industry and Countermeasures--A Study on the Development Mode of China's Sports Industry in the Background of “Internet +”. |
| 50 | 2023 | How Sports Consumption Impact the Development of Cities Under the New Development Paradigm-Empirical Analysis Based on the Panel Data of Jiangsu Province. |
| 57 | 2022 | The analysis of the impact of “Internet +” on the high-quality development of China's manufacturing industry. |
| 58 | 2022 | Impact of Digital Inclusive Finance on Rural Residents' Consumption-Based on Dynamic Effect and Threshold Effect. |

**Q14** In summarizing your conclusion and discussion interpret your hypotheses and problem statement with evidence from your literature review section and give logical reasoning that what you have claimed is in fact true.

**Response:** Thank you for your comment. We do believe that the discussion and conclusion sections of the study should correspond to the previous literature so as to strengthen the coherence of the article and enhance the theoretical foundation for the conclusions. Therefore, we have also cited the literature results in the previous section to support the discussion sections, and listed them in the table as follows:

| No. | Title |
| --- | --- |
| 18 | The Role and Strategy of Sport Industry to Boost Economic Powerhouse Construction in the New Era |
| 19 | The Association Effect of Sports Industry on Economy And Its Regional Heterogeneity—Analysis of Provincial Panel Data Based on Stata |
| 43 | Sport and social media research: A review |
| 34 | The Research on the Change of Sports Product Consumption Model Under the Background of "Internet +" |

**Q15** The limitations and policy implications should be discussed in the conclusion section.

**Response:** Thank you for your comment. To be more rigorous, we modified the structure of the discussion and conclusion section. We moved the policy implication from the discussion section to the second paragraph of the conclusion section.
