# Supplementary material for: An analysis of the leverage effect of “Internet +” on the economic spillover of sports industry
Source: PLoS One. 2023 Jul 19;18(7):e0288778. doi: 10.1371/journal.pone.0288778 (PMC10355464; doi:10.1371/journal.pone.0288778)
Supplement: Peer review history

# Peer review: Decision Letter 0

**DOI:** 10.1371/journal.pone.0288778.r001

**Authors:** Uzair Aslam Bhatti

28 Dec 2022

PONE-D-22-32398An analysis of the leverage effect of “Internet +” on the economic spillover of sports industryPLOS ONE

Dear Dr. Yi,

Thank you for submitting your manuscript to PLOS ONE. After careful consideration, we feel that it has merit but does not fully meet PLOS ONE’s publication criteria as it currently stands. Therefore, we invite you to submit a revised version of the manuscript that addresses the points raised during the review process.

Please submit your revised manuscript by Feb 11 2023 11:59PM. If you will need more time than this to complete your revisions, please reply to this message or contact the journal office at plosone@plos.org. When you're ready to submit your revision, log on to https://www.editorialmanager.com/pone/ and select the 'Submissions Needing Revision' folder to locate your manuscript file.

Please include the following items when submitting your revised manuscript:

- A rebuttal letter that responds to each point raised by the academic editor and reviewer(s). You should upload this letter as a separate file labeled 'Response to Reviewers'.
- A marked-up copy of your manuscript that highlights changes made to the original version. You should upload this as a separate file labeled 'Revised Manuscript with Track Changes'.
- An unmarked version of your revised paper without tracked changes. You should upload this as a separate file labeled 'Manuscript'.

If you would like to make changes to your financial disclosure, please include your updated statement in your cover letter. Guidelines for resubmitting your figure files are available below the reviewer comments at the end of this letter.

If applicable, we recommend that you deposit your laboratory protocols in protocols.io to enhance the reproducibility of your results. Protocols.io assigns your protocol its own identifier (DOI) so that it can be cited independently in the future. For instructions see: https://journals.plos.org/plosone/s/submission-guidelines#loc-laboratory-protocols. Additionally, PLOS ONE offers an option for publishing peer-reviewed Lab Protocol articles, which describe protocols hosted on protocols.io. Read more information on sharing protocols at https://plos.org/protocols?utm_medium=editorial-email&utm_source=authorletters&utm_campaign=protocols.

We look forward to receiving your revised manuscript.

Kind regards,

Uzair Aslam Bhatti

Academic Editor

PLOS ONE

Journal Requirements:

When submitting your revision, we need you to address these additional requirements.

1. Please ensure that your manuscript meets PLOS ONE's style requirements, including those for file naming. The PLOS ONE style templates can be found at 

https://journals.plos.org/plosone/s/file?id=wjVg/PLOSOne_formatting_sample_main_body.pdf and 

https://journals.plos.org/plosone/s/file?id=ba62/PLOSOne_formatting_sample_title_authors_affiliations.pdf

2. We note that the grant information you provided in the ‘Funding Information’ and ‘Financial Disclosure’ sections do not match. 

When you resubmit, please ensure that you provide the correct grant numbers for the awards you received for your study in the ‘Funding Information’ section.

3. Thank you for stating the following financial disclosure: 

"The author(s) received no specific funding for this work"

At this time, please address the following queries:

a) Please clarify the sources of funding (financial or material support) for your study. List the grants or organizations that supported your study, including funding received from your institution. 

b) State what role the funders took in the study. If the funders had no role in your study, please state: “The funders had no role in study design, data collection and analysis, decision to publish, or preparation of the manuscript.”

c) If any authors received a salary from any of your funders, please state which authors and which funders.

d) If you did not receive any funding for this study, please state: “The authors received no specific funding for this work.”

Please include your amended statements within your cover letter; we will change the online submission form on your behalf.

4. In your Data Availability statement, you have not specified where the minimal data set underlying the results described in your manuscript can be found. PLOS defines a study's minimal data set as the underlying data used to reach the conclusions drawn in the manuscript and any additional data required to replicate the reported study findings in their entirety. All PLOS journals require that the minimal data set be made fully available. For more information about our data policy, please see http://journals.plos.org/plosone/s/data-availability.

Upon re-submitting your revised manuscript, please upload your study’s minimal underlying data set as either Supporting Information files or to a stable, public repository and include the relevant URLs, DOIs, or accession numbers within your revised cover letter. For a list of acceptable repositories, please see http://journals.plos.org/plosone/s/data-availability#loc-recommended-repositories. Any potentially identifying patient information must be fully anonymized.

Important: If there are ethical or legal restrictions to sharing your data publicly, please explain these restrictions in detail. Please see our guidelines for more information on what we consider unacceptable restrictions to publicly sharing data: http://journals.plos.org/plosone/s/data-availability#loc-unacceptable-data-access-restrictions. Note that it is not acceptable for the authors to be the sole named individuals responsible for ensuring data access.

We will update your Data Availability statement to reflect the information you provide in your cover letter.

5. We note that you have stated that you will provide repository information for your data at acceptance. Should your manuscript be accepted for publication, we will hold it until you provide the relevant accession numbers or DOIs necessary to access your data. If you wish to make changes to your Data Availability statement, please describe these changes in your cover letter and we will update your Data Availability statement to reflect the information you provide.

**Additional Editor Comments:**

**​**Based on reviewer suggestion my decision is major revision.

[Note: HTML markup is below. Please do not edit.]

Reviewers' comments:

Reviewer's Responses to Questions

**Comments to the Author**

1. Is the manuscript technically sound, and do the data support the conclusions?

The manuscript must describe a technically sound piece of scientific research with data that supports the conclusions. Experiments must have been conducted rigorously, with appropriate controls, replication, and sample sizes. The conclusions must be drawn appropriately based on the data presented. 

Reviewer #1: Yes

Reviewer #2: Yes

**********

2. Has the statistical analysis been performed appropriately and rigorously? 

Reviewer #1: Yes

Reviewer #2: Yes

**********

3. Have the authors made all data underlying the findings in their manuscript fully available?

The PLOS Data policy requires authors to make all data underlying the findings described in their manuscript fully available without restriction, with rare exception (please refer to the Data Availability Statement in the manuscript PDF file). The data should be provided as part of the manuscript or its supporting information, or deposited to a public repository. For example, in addition to summary statistics, the data points behind means, medians and variance measures should be available. If there are restrictions on publicly sharing data—e.g. participant privacy or use of data from a third party—those must be specified.

Reviewer #1: Yes

Reviewer #2: Yes

**********

4. Is the manuscript presented in an intelligible fashion and written in standard English?

PLOS ONE does not copyedit accepted manuscripts, so the language in submitted articles must be clear, correct, and unambiguous. Any typographical or grammatical errors should be corrected at revision, so please note any specific errors here.

Reviewer #1: Yes

Reviewer #2: Yes

**********

5. Review Comments to the Author

Please use the space provided to explain your answers to the questions above. You may also include additional comments for the author, including concerns about dual publication, research ethics, or publication ethics. (Please upload your review as an attachment if it exceeds 20,000 characters)

Reviewer #1: The author explores the leverage effect of economic spillover of sports industry in the context of “Internet +”, examines the dynamic impact of “Internet +” on the economic spillover of sports industry through empirical research. Although the paper provide significant knowledge, however i have following concern in the paper.

1- The novelty of the paper is not validated by the authors. The paper should include a section in the introduction about the contribution of the paper as well as update the abstract.

2- The related work does not support the problem identified in this study. There should be citations for the statistical figures reported in the paper. This literature review should include recent citations from 2020, 2021, and 2022.

>https://doi.org/10.3390/atmos12101338

>https://doi.org/10.1109/CSDE50874.2020.9411587

>https://doi.org/10.1109/ICECube53880.2021.9628315

>https://doi.org/10.15244/pjoes/142146

>https://doi.org/10.3389/fenvs.2022.945628

>https://doi.org/10.3390/su13084312

3- The authors should consider the comparison of various existing studies in tabular form to validate the missing gap.

4- The author should provide an analysis of the proposed study. Analyses can be based on the same methods as the above papers.

5- Using benchmarks defined in relevant research, the author should consider a one-to-one comparison of the existing model and the proposed model.

6- The author should identify future research directions.

Reviewer #2: The study investigated the leverage effect of “Internet +” on the economic spillover of sports industry. The research design and methodology are proper. However, I think the introduction and discussion should be restructured and largely rewritten.

Keywords: Please add keywords.

Introduction

Please add references in the first few lines of the 1st paragraph.

Please clearly mention the uniqueness of your study in the first paragraph.

The 1st paragraph is extra large I suggest to split into two paragraphs and make it logical.

Discussion

Compare your result with previous similar studies.

Please mention future work in the conclusion.

Please add the conclusion part.

Reference

Please add the latest reference, especially the paper published in the last five years.

**********

6. PLOS authors have the option to publish the peer review history of their article (what does this mean?). If published, this will include your full peer review and any attached files.

If you choose “no”, your identity will remain anonymous but your review may still be made public.

**Do you want your identity to be public for this peer review?** For information about this choice, including consent withdrawal, please see our Privacy Policy.

Reviewer #1: No

Reviewer #2: **Yes: **Mir Muhammad Nizamani

**********

[NOTE: If reviewer comments were submitted as an attachment file, they will be attached to this email and accessible via the submission site. Please log into your account, locate the manuscript record, and check for the action link "View Attachments". If this link does not appear, there are no attachment files.]

While revising your submission, please upload your figure files to the Preflight Analysis and Conversion Engine (PACE) digital diagnostic tool, https://pacev2.apexcovantage.com/. PACE helps ensure that figures meet PLOS requirements. To use PACE, you must first register as a user. Registration is free. Then, login and navigate to the UPLOAD tab, where you will find detailed instructions on how to use the tool. If you encounter any issues or have any questions when using PACE, please email PLOS at figures@plos.org. Please note that Supporting Information files do not need this step.

# Author comment: Author response to Decision Letter 0

**DOI:** 10.1371/journal.pone.0288778.r002

10 Feb 2023

On behalf of all the contributing authors, I would like to express our sincere appreciation for your constructive comments concerning our article. These comments are all valuable and helpful for improving our article. According to your comments, we have extensively modified our manuscript to make our results more convincing. In this revised version, our manuscript's changes were highlighted within the document using tracked changes in red. We offer a detailed point-by-point response to your insightful questions and suggestions below.

Reviewer #1: 

Q1. The novelty of the paper is not validated by the authors. The paper should include a section in the introduction about the contribution of the paper as well as update the abstract.

Response: Thank you for this comment and suggestion; we do need to include the a section in the introduction about the contribution of the paper as well as update the abstract. Therefore, we rewrote the last paragraph of the introduction and updated the abstract.

Abstract

Given the unprecedented and profound impact on the traditional sports industry and its economic driving force brought by the “Internet +” , the article analyzes the driving relationship between the sports industry and local economic development and deconstructs the dynamic regulation mechanism of “Internet +” on the economic spillover of the sports industry. Empirical research based on inter-provincial panel data reveals that the leverage effect of economic spillover of the sports industry has significantly promoted regional economic growth, and “Internet +” functions as a two-way regulator. Only when the development level of the Internet exceeds a certain threshold can “Internet +” form an efficacious complementary mechanism with the sports industry. The economic spillover dividend of the sports industry will accelerate regional economic growth. However, the impact of “Internet +” on the threshold regulation of the sports industry economic spillover has spatial and temporal differentiation characteristics in the three dimensions of Internet hardware level, Internet penetration rate, and Internet application value. Therefore, it is required to apply differential regulation strategies in future policy design to realize the integration-driven and empower the high-quality development of “Internet+” and digital sports.

Introduction

The Internet has entered all circles of people’s lives, but its impact on the economic spillover effect of the sports industry is still uncertain. Therefore, how to stimulate the economic spillover effect of the sports industry in the context of the Internet is of great theoretical value and practical significance. This study concentrates on the economic spillover effect of the sports industry and utilizes China’s inter-provincial panel data from 2015-2019 to deconstruct the mechanism of the Internet. The purpose of this study was to: (1) explore the leverage effect of economic spillover of the sports industry in the context of “Internet +”; (2) examine what the specific regulation mechanism is; (3) investigate the practical needs of transformation of the sports industry and the dilemmas during the process of transformation. This study offers new insights into the role of the Internet in the process of the sports industry driving the regional economy and suggests future directions for sports industry research and government policies.

Q2. The related work does not support the problem identified in this study. There should be citations for the statistical figures reported in the paper. This literature review should include recent citations from 2020, 2021, and 2022.

Response: Thank you for this comment and examples, indeed, we also deeply believe that our manuscript should include more citations for the statistical figures from 2020, 2021, and 2022. Therefore, we added following citations in red in our manuscript. 

(1)Line 117, page 6: In the study on the interaction between the sports industry and regional economic development, Zhang Liang noted that when the per capita disposable income (PCDI) of urban residents is less than 13659.26 yuan, increasing investment in the sports industry is not conducive to regional economic growth; in areas where the PCDI is between 13659.26 and 21168.79 yuan, the effect of investment in the sports industry on regional economic growth is not significant; when the PCDI exceeds 21168.79 yuan, increasing investment will significantly contribute to regional economic growth [28].

(2)Line 127, page 7: The added value of the sports industry is an important indicator that can reflect the development level of the sports industry. For example, from the data of 2017, the added value of the sports industry in Fujian, Guangdong, and Jiangsu in the eastern region is 132.401 billion yuan, 132.186 billion yuan, and 121.955 billion yuan, respectively. At the same time, the added value of the sports industry in Henan, Hunan, and Anhui in the central region reveals that the level of regional economic development not only directly affects the results but also determines the ability of their sports industry development [30].

(3)Line 133, page 7: The coupling degree between the sports industry and economic development in Shanghai in the eastern region was 0.4986, 0.4965, and 0.4992 from 2015 to 2017, respectively, and the coupling degree between the sports industry and economic development in other provinces during the same period was no higher than 0.5. The results reflect that the sports industry has been improving its quality and efficiency in recent years, with an average annual growth rate higher than that of GDP in the same period. However, the regional economy and sports industry system is a contradictory and unified; the two complement each other and promote each other to produce a combined effect, achieving sustainable and joint development [31]. 

(4)Line 153, page 8: By applying “internet+” to the sports industry, the innovative development of new formats such as Internet broadcasting, intelligent software and hardware, e-commerce, and esports can be realized. For instance, the revenue of sports software has increased from 108 million yuan in 2010 to 352 million yuan in 2021 [38].

(5)Line 170, page 8. The transformation of the sales of sports products in the context of “Internet +” has revolutionized the process of sports consumption, with material consumption such as sports equipment and apparel, and service consumption such as sports events and training changing to O2O (Online to Offline) mode, meanwhile, “Internet +” has also profoundly affected the consumption style and process of products including sports lottery, esports, sports copyright, physical education, and scientific research, and culture [42], for instance, according to the data of Taiwan Sports Lottery from May 1, 2018 to April 30, 2019, the betting rate with mobile phone and tablet computer was as high as 70% [43]. 

(6)Line 179, page 9. The shift of consumption characteristics in the context of “Internet +” has reconstructed the form of economic rents obtained by enterprises. In the age of Internet 2.0, the sharing model has become prevailing. The connecting dividend rental has gradually replaced the ability rental under the role of the Internet so that enterprises no longer make money by directly selling products, nor do they concentrate on chasing product sales dividends. Thus a great number of traditional fitness companies have already started to utilize their offline resources to enhance digital upgrades[44]. The shift was even more prominent after the outbreak. An analysis of 251 people revealed that 67.7% of respondents were not interested in visiting offline retail channels in the future [45]. 

Q3. The authors should consider the comparison of various existing studies in tabular form to validate the missing gap.

Response: Thank you for this comment, and we do believe that the tabular form can validate the missing gap. Therefore we added a form in the commentary part as follows: 

Table 1: Different Research on Sports and Regional Economy

Region/Scope Context Research Methods Research Subjects

National None Quantitative Core elements for coordinated development of regional economy and sports industry [22]. 

12 Provinces and Cities None Quantitative The coupling degree and coordination degree of sports industry and regional economy [29].

National China’s New Era Qualitative The strategy of the sports industry contributes to the construction of economic power. The sports industry is conductive to the ecological economy of regional development [25].

12 Provinces and Cities None Quantitative The relationship and heterogeneity of sports industry and regional economy [24].

12 Provinces and Cities None Quantitative Regional characteristics of the sports industry development [28].

14 Provinces and Cities Digital Economy Quantitative the Impact of Digital Economy on the High-Quality Development of Sports Industry.[49]

Q4. The author should provide an analysis of the proposed study. Analyses can be based on the same methods as the above papers.

Response: Thank you for this comment, and we do reckon that adding an analysis of the proposed study would contribute more credibility to our study. Therefore, we added a similar study that applied the same data and similar methods in the commentary section. We rewrote the paragraph as follows: 

As seen in Table 1, most Chinese scholars stated that the sports industry could assist in developing the regional economy. Some studies use the same database as this paper to analyze the correlation between the sports industry and regional economy and put forward the concepts of regional heterogeneity, coupling degree, and evaluation index, which provide rich research ideas and a theoretical basis for the study of the sports industry and regional economy. For instance, An analysis applied a time-fixed effects model to study the economic growth and sports industry structure in 12 provinces and cities in different regions of the country. Controlling for other variables, the total output and value-added of the sports industry were significant at the 1% level and contributed significantly to regional GDP. Most Chinese scholars stated that the sports industry could assist the development of the regional economy. Some of them analyzed the correlation between the sports industry and the regional economy, and researches on the impact of the sports industry on the regional economy is more abundant. The multi-dimensional investigation is combined with the industrial characteristics and geographical location, which provides a wealth of research ideas and theoretical foundations for the research on the sports industry and regional economy. However, it can be seen that there exist specific differences in the industrial development stage, industrial pattern, and market environment in China’s sports industry in different regions. Therefore, the results from a nationwide study can not fully represent the relationship between the sports industry and the regional economy. 

In addition, in the background of industrial networking, domestic researchers have focused on the prospect and strategy of sports industry development under the context of “Internet +”, and a comprehensive analysis reveals that “Internet +” influences not only the sports business market but also the whole sports industry ecology, and the development of sports industry under the context of “Internet +” in China is still in the primary stage. The studies are mainly based on qualitative theoretical deduction research, which lacks objective quantitative research support, thus hindering the expansion of research profundity to a certain extent. This paper intends to analyze the driving relationship between the sports industry and regional economic development, explore the profound and intrinsic attributes of the sports industry in the context of “Internet +”, and deconstruct the dynamic regulation mechanism of Internet +” on the economic spillover of the sports industry by applying statistics and econometric model, so as to promote the integration and drive of “Internet +” and digital sports, and empower high-quality development in China.

Q5. Using benchmarks defined in relevant research, the author should consider a one-to-one comparison of the existing model and the proposed model.

Response: Thank you for your comments and suggestion, and we believed it’s more convincing to present a comparison of the existing model and the proposed model. Therefore, we corrected the econometric model part and list the advantages of proposed model as follows:

In this paper, we applied Hanse’s panel threshold regression model. Previous research utilized group testing or constructed multiplicative estimation models to analyze nonlinear threshold characteristics. However, both methods have certain shortcomings. First, it is difficult to solidify the grouping criteria in group testing, which may distort threshold estimation and lack of corresponding significance test. The multiplicative estimation model can estimate the overall impact threshold, but the cross-term cannot effectively analyze the impact relationship between core explanatory variables and threshold variables. The estimation results have limitations in interpretation. Hansen’s panel threshold regression model effectively compensates for the technical shortcomings of the above two threshold tests by constructing piecewise functions of the regression coefficients of the explanatory variables, which can not only estimate the thresholds but also further test the reasonableness of the thresholds for significance.

Q6. The author should identify future research directions.

Response: Thank you for your careful reading and suggestion, and we proposed future research directions in the conclusion part as follows: 

Theoretically and practically, this study provides some meaningful insights for future research work. From a theory perspective, there are questions that need to be further verified in future studies, for instance, whether the relationship between the sports industry and regional economy will be moderated by other confounding factors, whether the results will remain the same with statistics from other provinces and cities, and whether threshold regulation variables can fully reflect the level of the Internet development. From a practical perspective, more quantitative cohort research is needed to analyze the Internet's influence and specific functional mechanism on the sports industry and regional economic development. Second, the transformation and upgrading of the sports industry through the digital economy have become a trend. As a result, more and more sports enterprises will invest more in Internet applications, digital sports security, and digital sports talents, and Meta plus sports will become an essential research direction in the future. Third, bringing prosperity to all is an essential requirement of socialism with Chinese characteristics, and well-balanced development is one of the premier goals of China’s development. Therefore, future research should focus on achieving a balanced development of the Internet in different regions in terms of Internet construction level, Internet penetration rate, and commercial Internet application, and thus promote the balanced development of the sports industry and economy and mitigate the heterogeneity in different regions.

Reviewer #2: 

Q1. Keywords: Please add keywords.

Response: Thank you for your careful reading and rigorous attitude, and we are really sorry for our careless mistakes. We have added the keywords in the manuscript as follows: 

Keywords: sports industry, “Internet +”, economic spillover, leverage effect, digital sports

Q2. Introduction: Please add references in the first few lines of the 1st paragraph.

Response: Thank you for this comment, and we believe adding references in the first few lines of the 1st paragraph is more convincing. Thus, we added the following references.

Entering the new era, the role of China's sports industry in the national economic and social development has become increasingly prominent [1], which has become a new growth point of the national economy, and the sports industry plays a highly significant role in facilitating the construction of an economic power, the construction of Healthy China and the construction of a sports power [2]. At the third session of the 12th National People’s Congress, the “Internet +” action program was first proposed by Premier Li Keqiang in the government work report [3].

[1] Ren B, Huang H. Practical Problems and Development Strategies of China's Sports Industry Under the Background of Sporting Powerhouse Construction. Sports Culture Guide. 2022; (04):68-74+89. doi:CNKI:SUN:TYWS.0.2022-04-011.

[2] Huang H, Kang L. heoretical Logic and Implementation Path of High-Quality Development of Sports Industry in the New Era.China Sport Science. 2022; 42(01):15-34+58.DOI:10.16469/j.css.202201002.

[3] Li K. REPORT ON THE WORK OF THE GOVERNMENT-Delivered at the Third Session of the 12th National People’s Congress of the People’s Republic of China on March 5, 2015. Available from: http://www.gov.cn/guowuyuan/2015-03/16/content_2835101.htm (accessed 24th January 2023)

Q3. Introduction: Please clearly mention the uniqueness of your study in the first paragraph.

Response: Thank you for your careful reading, and we do believe that it will be more clear and understandable to add the uniqueness in the first paragraph. Thus, we added the following in the first paragraph of the introduction part.

The downward pressure on China’s economic growth is rising continuously, and the increasing trend of the sports industry can not only hedge the risk to some extent but also contribute to industrial restructuring. Therefore, this study concentrates on analyzing the relationship between the sports industry and regional economic development and how to maximize the spillover effect of the sports industry to promote the regional economy’s development better.

Q4. Introduction: The 1st paragraph is extra large I suggest to split into two paragraphs and make it logical.

Response: Thank you for your careful reading, and we rewrote the first paragraph and split it into two paragraphs logically as follows: 

Entering the new era, the role of China's sports industry in the national economic and social development has become increasingly prominent [1], which has become a new growth point of the national economy, and the sports industry plays a highly significant role in facilitating the construction of an economic power, the construction of Healthy China and the construction of a sports power [2]. At the third session of the 12th National People’s Congress, the “Internet +” action program was first proposed by Premier Li Keqiang in the government work report [3]. The sports industry in China maintained a torrid growth during 2016-2019, as the total scale increased beyond a trillion yuan, up by 55.1%, which is 1.14% of GDP surged from 0.87%. 

Due to the Covid-19 pandemic, the speed of economic growth of the national value-added of the tertiary industry slowed sharply in 2020 [4]. While the sports industry transformed to digital sports with the help of new technologies, such as big data[5], “Internet +” [6], 5G [7], etc., the value-added of sports media and information service industry maintained 18.9% growth, and the value-added of physical education and training industry expanded 5.7% through new business models like livestreaming fitness[8] and online training [9]. The sports industry’s economic effect in China is getting gradually prominent. The downward pressure on China's economic growth is rising continuously, and the increasing trend of the sports industry can not only hedge the risk to some extent but also contribute to industrial restructuring. Therefore, this study concentrates on analyzing the relationship between the sports industry and regional economic development and how to maximize the spillover effect of the sports industry to promote the regional economy's development better.

Q5. Discussion: Compare your result with previous similar studies

Response: Thank you for your comments, and we all consider it necessary to list comparisons. Therefore, we rewrote the first paragraph of the discussion part as follows: 

In this study, we applied China’s inter-provincial panel data from 2015-2019 to examine the driving relationship between the sports industry development level and regional economic development and further explore the economic spillover impact of "Internet +" on the sports industry. Similar to a previous study on the relationship and heterogeneity between the sports industry and the regional economy [26], this study found a significant correlation between the sports industry and the regional economy. However, this study also verified a positive relationship of dependence between the impact of the sports industry on regional economic growth and the regulation mechanism of the Internet. Compared to empirical research on the impact of the digital economy on sports development which reveals that the digital economy is positive for the high-quality development of the sports industry at a significance level of 1% [49], this study signifies that the regulating effect of the Internet on the economic spillover of the sports industry has complex non-linear characteristics. Only when the level of Internet development exceeds a specific threshold can it develop a benign complementary mechanism with the sports industry, leveraging the economic spillover dividends of the sports industry and creating new economic growth points. As the previous study reveals, regional heterogeneity exists in the sports industry’s impact on the regional economy. This study also found regional heterogeneity in the regulation effect of the Internet. The core problem is that each province’s Internet development level is difficult to coordinate in the three dimensions of Internet construction level, Internet penetration rate, and Internet business application value.

Q6. Please mention future work in the conclusion.

Response: Thank you for your comment and suggestion, and we highly agree that it is necessary to propose future research directions in the conclusion part. Therefore, we added future research directions in the conclusion part as follows: 

Theoretically and practically, this study provides some meaningful insights for future research work. From a theory perspective, there are questions that need to be further verified in future studies, for instance, whether the relationship between the sports industry and regional economy will be moderated by other confounding factors, whether the results will remain the same with statistics from other provinces and cities, and whether threshold regulation variables can fully reflect the level of the Internet development. From a practical perspective, more quantitative cohort research is needed to analyze the Internet's influence and specific functional mechanism on the sports industry and regional economic development. Second, the transformation and upgrading of the sports industry through the digital economy have become a trend. As a result, more and more sports enterprises will invest more in Internet applications, digital sports security, and digital sports talents, and Meta plus sports will become an essential research direction in the future. Third, bringing prosperity to all is an essential requirement of socialism with Chinese characteristics, and well-balanced development is one of the premier goals of China’s development. Therefore, future research should focus on achieving a balanced development of the Internet in different regions in terms of Internet construction level, Internet penetration rate, and commercial Internet application, and thus promote the balanced development of the sports industry and economy and mitigate the heterogeneity in different regions.

Q7. Please add the conclusion part.

Response: Thank you for your comment and suggestion, and we have added the the conclusion part in the revised manuscript as follows:

Conclusion

This paper revealed a significant correlation between the sports industry and the regional economy. Furthermore, it verified a positive relationship of dependence between the impact of the sports industry on regional economic growth and the regulation mechanism of the Internet. However, the regulating effect of the Internet on the economic spillover of the sports industry has complex non-linear characteristics. Only when the level of Internet development exceeds a specific threshold can it develop a benign complementary mechanism with the sports industry. Internet construction level, penetration rate, and business application value are three dimensions contributing to the regional heterogeneity of regulation effects. According to the results, this study also promotes suggestions for future policies. The sports industry’s development follows the market economy development principle, and the generated marginal consumption has already expanded market space. Thus, it is a practical choice for local governments to develop the economy through the sports industry. Moreover, the “Internet +” is the binding force for transforming and upgrading the sports industry in the digital economy era. Therefore, it is necessary for provinces and cities to enhance the Internet penetration rate, expand Internet business applications, and upgrade the level of Internet development to a specific point to maximize the drive of positive spillover. The regional heterogeneity nature requires more concentration on the middle and western regions.

Theoretically and practically, this study provides some meaningful insights for future research work. From a theory perspective, there are questions that need to be further verified in future studies, for instance, whether the relationship between the sports industry and regional economy will be moderated by other confounding factors, whether the results will remain the same with statistics from other provinces and cities, and whether threshold regulation variables can fully reflect the level of the Internet development. From a practical perspective, more quantitative cohort research is needed to analyze the Internet's influence and specific functional mechanism on the sports industry and regional economic development. Second, the transformation and upgrading of the sports industry through the digital economy have become a trend. As a result, more and more sports enterprises will invest more in Internet applications, digital sports security, and digital sports talents, and Meta plus sports will become an essential research direction in the future. Third, bringing prosperity to all is an essential requirement of socialism with Chinese characteristics, and well-balanced development is one of the premier goals of China’s development. Therefore, future research should focus on achieving a balanced development of the Internet in different regions in terms of Internet construction level, Internet penetration rate, and commercial Internet application, and thus promote the balanced development of the sports industry and economy and mitigate the heterogeneity in different regions.

Attachment Submitted filename: Response to Reviewers.docx Click here for additional data file.

# Peer review: Decision Letter 1

**DOI:** 10.1371/journal.pone.0288778.r003

**Authors:** Shujahat Haider Hashmi

9 May 2023

PONE-D-22-32398R1An analysis of the leverage effect of “Internet +” on the economic spillover of sports industryPLOS ONE

Dear Dr. Yi,

Thank you for submitting your manuscript to PLOS ONE. After careful consideration, we feel that it has merit but does not fully meet PLOS ONE’s publication criteria as it currently stands. Therefore, we invite you to submit a revised version of the manuscript that addresses the points raised during the review process.

You can see that  the reviewers have now reevaluated your research work and they have suggested some minor comments to improve the quality of the manuscript. Therefore, you are now directed to incorporate these changes and submit the revised version, along with point-by-point responses to reviewers' comments, within the due date.

Please submit your revised manuscript by Jun 23 2023 11:59PM. If you will need more time than this to complete your revisions, please reply to this message or contact the journal office at plosone@plos.org. When you're ready to submit your revision, log on to https://www.editorialmanager.com/pone/ and select the 'Submissions Needing Revision' folder to locate your manuscript file.

Please include the following items when submitting your revised manuscript:

- A rebuttal letter that responds to each point raised by the academic editor and reviewer(s). You should upload this letter as a separate file labeled 'Response to Reviewers'.
- A marked-up copy of your manuscript that highlights changes made to the original version. You should upload this as a separate file labeled 'Revised Manuscript with Track Changes'.
- An unmarked version of your revised paper without tracked changes. You should upload this as a separate file labeled 'Manuscript'.

If you would like to make changes to your financial disclosure, please include your updated statement in your cover letter. Guidelines for resubmitting your figure files are available below the reviewer comments at the end of this letter.

If applicable, we recommend that you deposit your laboratory protocols in protocols.io to enhance the reproducibility of your results. Protocols.io assigns your protocol its own identifier (DOI) so that it can be cited independently in the future. For instructions see: https://journals.plos.org/plosone/s/submission-guidelines#loc-laboratory-protocols. Additionally, PLOS ONE offers an option for publishing peer-reviewed Lab Protocol articles, which describe protocols hosted on protocols.io. Read more information on sharing protocols at https://plos.org/protocols?utm_medium=editorial-email&utm_source=authorletters&utm_campaign=protocols.

We look forward to receiving your revised manuscript.

Kind regards,

Shujahat Haider Hashmi, PhD Regional Economics

Academic Editor

PLOS ONE

Journal Requirements:

Please review your reference list to ensure that it is complete and correct. If you have cited papers that have been retracted, please include the rationale for doing so in the manuscript text, or remove these references and replace them with relevant current references. Any changes to the reference list should be mentioned in the rebuttal letter that accompanies your revised manuscript. If you need to cite a retracted article, indicate the article’s retracted status in the References list and also include a citation and full reference for the retraction notice.

Additional Editor Comments:

The reviewers have now reevaluated your research work and they have suggested some minor comments to improve the quality of the manuscript. You are now directed to incorporate these changes and submit the revised version within the due date.

[Note: HTML markup is below. Please do not edit.]

Reviewers' comments:

Reviewer's Responses to Questions

**Comments to the Author**

1. If the authors have adequately addressed your comments raised in a previous round of review and you feel that this manuscript is now acceptable for publication, you may indicate that here to bypass the “Comments to the Author” section, enter your conflict of interest statement in the “Confidential to Editor” section, and submit your "Accept" recommendation.

Reviewer #3: All comments have been addressed

Reviewer #4: All comments have been addressed

Reviewer #5: (No Response)

**********

2. Is the manuscript technically sound, and do the data support the conclusions?

The manuscript must describe a technically sound piece of scientific research with data that supports the conclusions. Experiments must have been conducted rigorously, with appropriate controls, replication, and sample sizes. The conclusions must be drawn appropriately based on the data presented. 

Reviewer #3: Yes

Reviewer #4: Yes

Reviewer #5: Yes

**********

3. Has the statistical analysis been performed appropriately and rigorously? 

Reviewer #3: Yes

Reviewer #4: Yes

Reviewer #5: Yes

**********

4. Have the authors made all data underlying the findings in their manuscript fully available?

The PLOS Data policy requires authors to make all data underlying the findings described in their manuscript fully available without restriction, with rare exception (please refer to the Data Availability Statement in the manuscript PDF file). The data should be provided as part of the manuscript or its supporting information, or deposited to a public repository. For example, in addition to summary statistics, the data points behind means, medians and variance measures should be available. If there are restrictions on publicly sharing data—e.g. participant privacy or use of data from a third party—those must be specified.

Reviewer #3: Yes

Reviewer #4: Yes

Reviewer #5: Yes

**********

5. Is the manuscript presented in an intelligible fashion and written in standard English?

PLOS ONE does not copyedit accepted manuscripts, so the language in submitted articles must be clear, correct, and unambiguous. Any typographical or grammatical errors should be corrected at revision, so please note any specific errors here.

Reviewer #3: Yes

Reviewer #4: Yes

Reviewer #5: Yes

**********

6. Review Comments to the Author

Please use the space provided to explain your answers to the questions above. You may also include additional comments for the author, including concerns about dual publication, research ethics, or publication ethics. (Please upload your review as an attachment if it exceeds 20,000 characters)

Reviewer #3: Thank you for considering given comments seriously and providing response comprehensively.

However this may add an ice to the cake if you make following changes/additions.

• Add a small paragraph at the end of introduction section and provide key findings.

• Also add the structure of paper soon after providing key findings.

• Adjust table 05 properly

Good luck.

Reviewer #4: I have reviewed the manuscript, comments, and responses, and while I find them satisfactory, I have a few suggestions to improve the clarity and coherence of the text.

Firstly, I noticed that the title suggests that "economic spillover" is the dependent variable (DV), as indicated by the keywords. However, the authors switch between the terms "economic development" and "economic growth", which have distinct meanings and measures and are not necessarily proxies for economic spillover. Therefore, I would recommend that the authors clarify and be consistent about what they are analyzing as the DV, and ensure that it is clearly stated in the title, keywords, and throughout the text.

Secondly, I would suggest that the authors consider adding a brief paragraph or two in the introduction section to summarize the policy implications and key results of their study. This would provide readers with a clear understanding of the potential real-world applications of the research and help to contextualize the findings.

Overall, these suggestions would help to improve the clarity and coherence of the manuscript, and ensure that readers have a clear understanding of the research aims and implications.

Reviewer #5: Abstract

1. The Abstract is the “Face” of the study, it must be high quality and informative. The abstract should be very specific to the aims, methodology, findings, and implications. Please focus the abstract on your study objectives and results. Results should be supported by their significance. Study implications should be discussed and based on the results.

Introduction

1. The paper lacks arguments on how and in what aspects it contributes to the literature. Objectives of the paper needs to be linked to the literature to identify how this paper is different than the previous ones and how it contributes to the literature on leverage effect of “Internet +” on the economic spillover of sports industry.

2. Justification of the topic is too weak, and not supported by the strong empirical work.

3. The author did not mention clearly about his/her contribution of the study, which should be included at the end of the introduction.

Literature review

1. A good literature review is included in the paper, but still no arguments can be seen in the selection of the study variables, theorization part in the literature review is weak.

2. More contextual review should be included.

Methodology

1. Why author has selected time period from 2015-2019? Provide rational for it.

2. Hanse’s panel threshold regression model is used for the study analysis, no argumentation/healthy discussion is provided about the suitability of this regression model.

3. Operationalization of the variables may be provided in tabular form.

Results

1. The author must provide the rationale for choosing the control variables such as Urbanization and trade.

2. The assumptions of Hanse’s model are not tested.

3. Results of the model are only discussed statistically but not supported by the previous literature.

4. The discussion is not based on latest literature. Support your discussion with recent literature.

5. In summarizing your conclusion and discussion interpret your hypotheses and problem statement with evidence from your literature review section and give logical reasoning that what you have claimed is in fact true.

6. The limitations and policy implications should be discussed in the conclusion section.

**********

7. PLOS authors have the option to publish the peer review history of their article (what does this mean?). If published, this will include your full peer review and any attached files.

If you choose “no”, your identity will remain anonymous but your review may still be made public.

**Do you want your identity to be public for this peer review?** For information about this choice, including consent withdrawal, please see our Privacy Policy.

Reviewer #3: No

Reviewer #4: No

Reviewer #5: No

**********

[NOTE: If reviewer comments were submitted as an attachment file, they will be attached to this email and accessible via the submission site. Please log into your account, locate the manuscript record, and check for the action link "View Attachments". If this link does not appear, there are no attachment files.]

While revising your submission, please upload your figure files to the Preflight Analysis and Conversion Engine (PACE) digital diagnostic tool, https://pacev2.apexcovantage.com/. PACE helps ensure that figures meet PLOS requirements. To use PACE, you must first register as a user. Registration is free. Then, login and navigate to the UPLOAD tab, where you will find detailed instructions on how to use the tool. If you encounter any issues or have any questions when using PACE, please email PLOS at figures@plos.org. Please note that Supporting Information files do not need this step.

Attachment Submitted filename: review-plos.docx Click here for additional data file.

# Author comment: Author response to Decision Letter 1

**DOI:** 10.1371/journal.pone.0288778.r004

25 May 2023

Dear Editor and reviewers:

On behalf of all the contributing authors, I would like to express our gratitude for your helpful feedback on our article. Your comments have been invaluable in improving our work. According to your suggestions, we have extensively modified our manuscript and added extra tables to make our results convincing. We have highlighted all the changes made to the manuscript using tracked changes in red. We offer a detailed point-by-point response to your insightful questions and suggestions below.

Reviewer #3: 

Q1&Q2 Add a small paragraph at the end of introduction section and provide key findings. Also add the structure of paper soon after providing key findings.

Response: Thank you for your valuable suggestion. We believe it would be clearer if we added the structure and key findings of the paper at the end of the introduction section. Therefore, we added the following paragraphs.

A linear estimation model with sports industry economic spillover as the dependent variable was designed based on the previous studies. The level of “Internet +” development was employed as the threshold regulation variable to examine its dynamic impact on the economic spillover of the sports industry. Inter-provincial panel data from 11 provinces and regions from 2015-2019 were collected and analyzed through the fixed-effects model, threshold model, robustness test, and heterogeneity analysis. This study enhances the current research by analyzing the sports economic spillover on the regional economy under the influence of the Internet. It supports the findings of Ruan’s study [17] and extends the theoretical study of Huang et al [18] by incorporating the Internet to explain the sports spillover effects on the regional economy through empirical analysis. Moreover, this study also validates the results of Jiang’s research [19] on the sports industry and regional economy heterogeneity.

The study finds a significant correlation between the sports industry and the regional economy. A positive relationship of dependence between the impact of the sports industry on regional economic development and the regulation mechanism of the Internet is significant, but the regulating effect of the Internet on the economic spillover of the sports industry has complex non-linear characteristics. Only when the level of Internet development exceeds a specific threshold can it develop a benign complementary mechanism with the sports industry. Based on the empirical results, this study suggests that it is a practical choice for local governments to develop the economy through the sports industry. As the “Internet +” is the binding force for transforming and upgrading the sports industry, provinces, and cities must enhance the Internet penetration rate, expand Internet business applications, and upgrade the level of Internet development to a specific point to maximize the drive of positive spillover. The regional heterogeneity nature requires more concentration on the middle and western regions.

Q3 Adjust table 05 properly

Response: Thank you for your suggestions. We are very sorry that we did not adjust Table 5 to meet the format required by the journal. Therefore, we have made proper adjustments to Table 6 in the revised manuscript (Table 5 in the original manuscript).

Reviewer #4: 

Q1 I noticed that the title suggests that “economic spillover” is the dependent variable (DV), as indicated by the keywords. However, the authors switch between the terms “economic development” and “economic growth”, which have distinct meanings and measures and are not necessarily proxies for economic spillover. Therefore, I would recommend that the authors clarify and be consistent about what they are analyzing as the DV, and ensure that it is clearly stated in the title, keywords, and throughout the text.

Response: Thank you for your suggestions. We do also believe that “economic spillover” is different from “economic development” and “economic growth”, and they can not simply replace each other. We are very sorry for the confusion caused by the choice of terms. We employed “economic spillover” to present the effect of the sports industry on the regional economy, and we also examined whether the Internet regulated this direct spillover effect. Therefore, to be more consistent and rigorous, we have standardized the terminology by integrating the terms “economic growth” and “economic development” and including the description of the spillover effect.

Q2 Secondly, I would suggest that the authors consider adding a brief paragraph or two in the introduction section to summarize the policy implications and key results of their study. This would provide readers with a clear understanding of the potential real-world applications of the research and help to contextualize the findings.

Response: Thank you for your precious suggestion. We also consider that it will be more friendly for readers, if we could summarize their study’s policy implications and key results in the introduction section. Therefore, we added the following paragraphs.

A linear estimation model with sports industry economic spillover as the dependent variable was designed based on the previous studies. The level of “Internet +” development was employed as the threshold regulation variable to examine its dynamic impact on the economic spillover of the sports industry. Inter-provincial panel data from 11 provinces and regions from 2015-2019 were collected and analyzed through the fixed-effects model, threshold model, robustness test, and heterogeneity analysis. This study enhances the current research by analyzing the sports economic spillover on the regional economy under the influence of the Internet. It supports the findings of Ruan’s study [17] and extends the theoretical study of Huang et al [18] by incorporating the Internet to explain the sports spillover effects on the regional economy through empirical analysis. Moreover, this study also validates the results of Jiang’s research [19] on the sports industry and regional economy heterogeneity.

The study finds a significant correlation between the sports industry and the regional economy. A positive relationship of dependence between the impact of the sports industry on regional economic development and the regulation mechanism of the Internet is significant, but the regulating effect of the Internet on the economic spillover of the sports industry has complex non-linear characteristics. Only when the level of Internet development exceeds a specific threshold can it develop a benign complementary mechanism with the sports industry. Based on the empirical results, this study suggests that it is a practical choice for local governments to develop the economy through the sports industry. As the “Internet +” is the binding force for transforming and upgrading the sports industry, provinces, and cities must enhance the Internet penetration rate, expand Internet business applications, and upgrade the level of Internet development to a specific point to maximize the drive of positive spillover. The regional heterogeneity nature requires more concentration on the middle and western regions.

Reviewer #5: 

Abstract

Q1 The Abstract is the “Face” of the study, it must be high quality and informative. The abstract should be very specific to the aims, methodology, findings, and implications. Please focus the abstract on your study objectives and results. Results should be supported by their significance. Study implications should be discussed and based on the results.

Response: Thank you for your suggestions. We are all highly agree that the abstract should be more concise and specific to the objectives and results. Therefore, we concentrated more on the objectives and results and rearranged the abstract in a structural framework as follows:

Given the unprecedented and profound impact on the traditional sports industry and its economic driving force brought by the “Internet +”, the purpose of this study is to explore the driving relationship between the sports industry and local economic development and deconstruct the dynamic regulation mechanism of “Internet +” on the economic spillover of the sports industry. Empirical data collected from China’s 11 inter-provincial panel data from 2015-2019 were analyzed through the fixed-effects and threshold models. The study finds that the economic spillover of the sports industry has significantly promoted regional economic development, and the regulation effect of the Internet has complex non-linear characteristics. Only when Internet development exceeds a specific threshold can it develop a benign complementary mechanism with the sports industry. The impact of “Internet +” on the threshold regulation of the sports industry economic spillover has spatial and temporal differentiation characteristics in the three dimensions of Internet hardware level, Internet penetration rate, and Internet application value. The results of this study extend the current study, indicating that it is a practical choice for local governments to develop the economy through the sports industry. Provinces and cities should enhance the Internet penetration rate, expand Internet business applications, and upgrade Internet development to a specific point to maximize the drive of positive spillover. Regional heterogeneity requires differential regulation strategies and more concentration on the middle and western regions.

Introduction

Q2 The paper lacks arguments on how and in what aspects it contributes to the literature. Objectives of the paper needs to be linked to the literature to identify how this paper is different than the previous ones and how it contributes to the literature on leverage effect of “Internet +” on the economic spillover of sports industry.

Response: Thank you for your valuable suggestion. Therefore, we added a description of the comparison of the results of this paper with other literature in the last part of the introduction, which also presents the additional contribution of this study to the literature. The added sections are as follows:

In page 4, line 104: A linear estimation model with sports industry economic spillover as the dependent variable was designed based on the previous studies. The level of “Internet +” development was employed as the threshold regulation variable to examine its dynamic impact on the economic spillover of the sports industry. Inter-provincial panel data from 11 provinces and regions from 2015-2019 were collected and analyzed through the fixed-effects model, threshold model, robustness test, and heterogeneity analysis. This study enhances the current research by analyzing the sports economic spillover on the regional economy under the influence of the Internet. It supports the findings of Ruan’s study [17] and extends the theoretical study of Huang et al [18] by incorporating the Internet to explain the sports spillover effects on the regional economy through empirical analysis. Moreover, this study also validates the results of Jiang’s research [19] on the sports industry and regional economy heterogeneity.

Q3 Justification of the topic is too weak, and not supported by the strong empirical work.

Response: Thank you for your comment. We also believe aspects of the empirical argumentation for this theme could still be strengthened. Since the domestic literature directly related to this study is scarce, especially the empirical quantitative studies, the argumentation on the topic may not be sufficiently demonstrated. We hope that the results of this study derived from the existing literature and data will support future in-depth studies.

Q4 The author did not mention clearly about his/her contribution of the study, which should be included at the end of the introduction.

Response: Thank you for your valuable suggestion, and we also think including the study’s contribution is necessary. Therefore, we add the following paragraphs at the end of the introduction section. 

In page 5, line 109: This study enhances the current research by analyzing the sports economic spillover on the regional economy under the influence of the Internet. It supports the findings of Ruan’s study [17] and extends the theoretical study of Huang et al [18] by incorporating the Internet to explain the sports spillover effects on the regional economy through empirical analysis. Moreover, this study also validates the results of Jiang’s research [19] on the sports industry and regional economy heterogeneity.

The study finds a significant correlation between the sports industry and the regional economy. A positive relationship of dependence between the impact of the sports industry on regional economic development and the regulation mechanism of the Internet is significant, but the regulating effect of the Internet on the economic spillover of the sports industry has complex non-linear characteristics. Only when the level of Internet development exceeds a specific threshold can it develop a benign complementary mechanism with the sports industry. Based on the empirical results, this study suggests that it is a practical choice for local governments to develop the economy through the sports industry. As the “Internet +” is the binding force for transforming and upgrading the sports industry, provinces, and cities must enhance the Internet penetration rate, expand Internet business applications, and upgrade the level of Internet development to a specific point to maximize the drive of positive spillover. The regional heterogeneity nature requires more concentration on the middle and western regions.

Literature review

Q5 A good literature review is included in the paper, but still no arguments can be seen in the selection of the study variables, theorization part in the literature review is weak.

Response: Thank you for your suggestion and comments. We have conducted some discussions on the selection of variables in the material and method section, and we also believe that it is essential to add a discussion on the selection of variables in the literature review section. Therefore, we added the following literature and statements in the literature review and material and method section, respectively, to enhance the theoretical part.

In page 11, line 260: For instance, a time-fixed effects model with the scale and value-added of regional sports industry output as the independent variable was applied to examine the economic growth and sports industry structure in 12 provinces and cities in different country regions. Controlling for other variables, the total output and value-added of the sports industry are significant at the 1% level and contribute significantly to regional GDP.

In page 12, line 264: Besides, Sun investigated the mechanism of sports consumption boosting urban economic development through panel data of major cities in Jiangsu Province, using the level of sports consumption as the core explanatory variable.

In page 12, line 277: For example, Ruan’s study employed a random effects model with the level of development of the digital economy as the explanatory variable to investigate its effect on the structure of the sports industry.

In page 15, line 337: Therefore, in the following section, we designed an econometric model with the sports industry development as the explanatory variable and the Internet development as the regulation variable to test the dynamic impact of “Internet +” on the economic spillover of the sports industry through empirical research.

Q6 More contextual review should be included.

Response: Thank you for your suggestion. Indeed, it would be more convincing, if we could add diverse contextual review in the study. Thus, we added literatures of different contexts to enrich our study, and listed them in Table 1. 

Methodology

Q7 Why author has selected time period from 2015-2019? Provide rational for it.

Response: Thank you for your recommendation. We all believe that it will be more convincing if we could provide the reason for selecting the research period. Therefore, we add the following in the Data sources and testing section.

In page 19, line 436: In 2014, the General Office of the State Council issued the Opinions on Promoting National Fitness, Sports Consumption, and High-Quality Development of the Sports Industry. Since then, China’s sports industry’s added value and growth rate have grown significantly. However, the rapid development of the sports industry was hampered by Coivd-19 pandemic in 2020. Therefore, this paper selects the research period from 2015-2019, which is more representative.

Q8 Hanse’s panel threshold regression model is used for the study analysis, no argumentation/healthy discussion is provided about the suitability of this regression model.

Response: Thank you for this comment, and we also deeply believe that it is necessary to include the discussion of selecting the regression model. Since we have promoted the advantage of Hanse’s panel threshold regression model compared to group testing or constructed multiplicative estimation models, we added the following to explain the suitability comprehensively.

In page 16 line 374: The model captures this nonlinear relationship when there is a threshold effect, i.e., when the relationship between the explanatory and explained variables changes significantly at a threshold point. The data utilized in the study are rather balanced and stable, and the sample size is relatively large, while this contributes to a more accurate estimation of the threshold and related parameters. Also, one of the purposes of this study was to investigate the threshold regulation effect of the Internet on the economic spillover of the sports industry. Therefore, this model fits the purpose and data requirements of this study.

Q9 Operationalization of the variables may be provided in tabular form.

Response: Thank you for this comment, and we do believe that it is necessary to provide the tabular form to present the operationalization of variables. Therefore, we add the following table (Table 2 in the revised manuscript) in the section of the measurement of variables.

Variables Operation Definition Indicator Measure

GDP the economic development level of the regions GDP growth rate Ratio

Sport the level of sports industry development the scale of regional sports industry output value Ratio

Internet-1 Internet construction level the distance of Internet lines laid in each province and region Ratio

Internet-2 Internet penetration rate the total number of Internet users in each region Ratio

Internet-3 Internet commercial application value commercial transaction amount of regional Internet data flow Ratio

Urbanization the level of urbanization the proportion of the urban population Ratio

Human human resources conditions the number of years of education per capital in the region Ratio

Trade the contribution of the trade the ratio of import and export volume to GDP in the region Ratio

Capital the total productive capital owned by an economy region’s capital stock in the current year Ratio

Results

Q10 The author must provide the rationale for choosing the control variables such as Urbanization and trade.

Response: Thank you for your suggestion, and we also think that it will be more convincing if we could add the explanation or choosing the control variables. Therefore, we rewrote the paragraph in the control variable part of measurement of variables and added four literatures to support the selection.

In page18, line 414: The selection of control variables should be based on theoretical foundations and previous studies, and those variables that may interfere with the correlation between explanatory and explained variables should be selected. According to the current literature, urbanization [53], trade [54], human capital [55], and capital stock [56] may affect the correlation between the explanatory and the explained variables. Therefore, to achieve unbiased test results and consider the measurement and operationalization of the variables, this study includes relevant control variables affecting economic development in the model. Specifically, these include: Urbanization, measured by the proportion of the urban population in the region; humans, reflected by the number of years of education per capita in the region; Trade, measured by the ratio of import and export volume to GDP in the region; and Capital is evaluated by the region’s capital stock in the current year and treated as a logarithm in the regression.

Q11 The assumptions of Hanse’s model are not tested.

Response: Thank you for your comment. We also believe that it is necessary to test the model, thus, we applied endogenous control and robustness tests in the study. In the linear estimation model, to reduce the endogenous disturbance, the study logarithmically processed the observations variables and tested the linear model through fixed effects by incremental control variables. Meanwhile, the study altered the regression method to test the robustness of the original model through a random effects model. In the dynamic threshold estimation model, this study lagged the explanatory variables by one order to exclude endogenous disturbances. At the same time, we adjusted the data by removing the extreme figure interference to confirm the credibility. Also, before selecting the model, the study employed the Hausman test, which needed to be specifically described. Therefore, to be more rigorous, the following were added to the first part of the result section: 

In page 21, line 468: Three models are usually available for panel data analysis: the mixed estimation model, fixed effects model, and random effects model. However, the differences in time and cross-sectional dimensions of the data in this paper are relatively slight, and the p-value of the Hausman test is not significant, i.e., an individual fixed effect exists. Therefore, this study applied the fixed-effects model to assess the linear relationship between the level of sports industry development and regional economic development. 

Q12 Results of the model are only discussed statistically but not supported by the previous literature.

Response: Thank you for your comment. We do agree that it would be more persuasive to discuss the results by combining statistical data and literature. Therefore, we rewrote the first paragraph of the discussion part as follows:

In page 33, line 696: In this study, we applied China’s inter-provincial panel data from 2015-2019 to examine the driving relationship between the sports industry development level and regional economic development and further explore the leverage effect of “Internet +” on the sports industry economic spillover. This study found a significant correlation between the sports industry and the regional economy, which is in consistent with previous studies [18, 50]. Similar to the result of Zhong [43] and Pan’s [34] study, that the integration of the sports industry and the Internet could boost China’s economic development, this study also verified a positive relationship of dependence between the impact of the sports industry on regional economic development and the regulation mechanism of the Internet. Compared to empirical research on the impact of the digital economy on sports development which reveals that the digital economy is positive for the high-quality development of the sports industry at a significance level of 1% [49], this study signifies that the regulating effect of the Internet on the economic spillover of the sports industry has complex non-linear characteristics. Only when the level of Internet development exceeds a specific threshold can it develop a benign complementary mechanism with the sports industry, leveraging the economic spillover dividends of the sports industry and creating new economic growth points. This Internet threshold effect exists not only for the sports industry spillover, but also for areas such as manufacturing industry [57], residential consumption [58]. Concerning the results of previous research, this study finds that regional heterogeneity exists in the sports industry’s impact on the regional economy [19] and the regulation effect of the Internet. The core problem for the regional heterogeneity is that each province’s Internet development level is difficult to coordinate in the three dimensions of Internet construction level, Internet penetration rate, and Internet business application value.

Q13 The discussion is not based on latest literature. Support your discussion with recent literature.

Response: Thank you for your comment. We also consider that recent literature should be added to the discussion section to improve the temporal validity of the study and to endorse the findings. Therefore, based on the original literature, we added some latest literature to the discussion section and listed them in the table as follows:

No. Year Title

49 2021 Problems in the Development of China's Sports Industry and Countermeasures--A Study on the Development Mode of China's Sports Industry in the Background of “Internet +”.

50 2023 How Sports Consumption Impact the Development of Cities Under the New Development Paradigm-Empirical Analysis Based on the Panel Data of Jiangsu Province.

57 2022 The analysis of the impact of “Internet +” on the high-quality development of China's manufacturing industry.

58 2022 Impact of Digital Inclusive Finance on Rural Residents' Consumption-Based on Dynamic Effect and Threshold Effect.

Q14 In summarizing your conclusion and discussion interpret your hypotheses and problem statement with evidence from your literature review section and give logical reasoning that what you have claimed is in fact true.

Response: Thank you for your comment. We do believe that the discussion and conclusion sections of the study should correspond to the previous literature so as to strengthen the coherence of the article and enhance the theoretical foundation for the conclusions. Therefore, we have also cited the literature results in the previous section to support the discussion sections, and listed them in the table as follows:

No. Title

18 The Role and Strategy of Sport Industry to Boost Economic Powerhouse Construction in the New Era

19 The Association Effect of Sports Industry on Economy And Its Regional Heterogeneity—Analysis of Provincial Panel Data Based on Stata

43 Sport and social media research: A review

34 The Research on the Change of Sports Product Consumption Model Under the Background of "Internet +"

Q15 The limitations and policy implications should be discussed in the conclusion section.

Response: Thank you for your comment. To be more rigorous, we modified the structure of the discussion and conclusion section. We moved the policy implication from the discussion section to the second paragraph of the conclusion section.

Attachment Submitted filename: Response to Reviewers.docx Click here for additional data file.

# Peer review: Decision Letter 2

**DOI:** 10.1371/journal.pone.0288778.r005

**Authors:** Shujahat Haider Hashmi

4 Jul 2023

An analysis of the leverage effect of “Internet +” on the economic spillover of sports industry

PONE-D-22-32398R2

Dear Dr. Yi,

We’re pleased to inform you that your manuscript has been judged scientifically suitable for publication and will be formally accepted for publication once it meets all outstanding technical requirements.

Within one week, you’ll receive an e-mail detailing the required amendments. When these have been addressed, you’ll receive a formal acceptance letter and your manuscript will be scheduled for publication.

An invoice for payment will follow shortly after the formal acceptance. To ensure an efficient process, please log into Editorial Manager at http://www.editorialmanager.com/pone/, click the 'Update My Information' link at the top of the page, and double check that your user information is up-to-date. If you have any billing related questions, please contact our Author Billing department directly at authorbilling@plos.org.

If your institution or institutions have a press office, please notify them about your upcoming paper to help maximize its impact. If they’ll be preparing press materials, please inform our press team as soon as possible -- no later than 48 hours after receiving the formal acceptance. Your manuscript will remain under strict press embargo until 2 pm Eastern Time on the date of publication. For more information, please contact onepress@plos.org.

Kind regards,

Shujahat Haider Hashmi, PhD Regional Economics

Academic Editor

PLOS ONE

Additional Editor Comments (optional):

The reviewers have now thoroughly evaluated your resubmitted manuscript and have expressed due satisfaction with your revised work. Therefore, we feel great pleasure to inform you that your paper is now accepted for possible publication in the journal. We appreciate your continued efforts and patience to improve the quality of the manuscript. We wish you best of luck for your future endeavors.

Reviewers' comments:

Reviewer's Responses to Questions

**Comments to the Author**

1. If the authors have adequately addressed your comments raised in a previous round of review and you feel that this manuscript is now acceptable for publication, you may indicate that here to bypass the “Comments to the Author” section, enter your conflict of interest statement in the “Confidential to Editor” section, and submit your "Accept" recommendation.

Reviewer #3: All comments have been addressed

Reviewer #4: All comments have been addressed

**********

2. Is the manuscript technically sound, and do the data support the conclusions?

The manuscript must describe a technically sound piece of scientific research with data that supports the conclusions. Experiments must have been conducted rigorously, with appropriate controls, replication, and sample sizes. The conclusions must be drawn appropriately based on the data presented. 

Reviewer #3: Yes

Reviewer #4: Yes

**********

3. Has the statistical analysis been performed appropriately and rigorously? 

Reviewer #3: Yes

Reviewer #4: Yes

**********

4. Have the authors made all data underlying the findings in their manuscript fully available?

The PLOS Data policy requires authors to make all data underlying the findings described in their manuscript fully available without restriction, with rare exception (please refer to the Data Availability Statement in the manuscript PDF file). The data should be provided as part of the manuscript or its supporting information, or deposited to a public repository. For example, in addition to summary statistics, the data points behind means, medians and variance measures should be available. If there are restrictions on publicly sharing data—e.g. participant privacy or use of data from a third party—those must be specified.

Reviewer #3: Yes

Reviewer #4: (No Response)

**********

5. Is the manuscript presented in an intelligible fashion and written in standard English?

PLOS ONE does not copyedit accepted manuscripts, so the language in submitted articles must be clear, correct, and unambiguous. Any typographical or grammatical errors should be corrected at revision, so please note any specific errors here.

Reviewer #3: Yes

Reviewer #4: Yes

**********

6. Review Comments to the Author

Please use the space provided to explain your answers to the questions above. You may also include additional comments for the author, including concerns about dual publication, research ethics, or publication ethics. (Please upload your review as an attachment if it exceeds 20,000 characters)

Reviewer #3: I have carefully reviewed the manuscript titled and would like to inform you that the authors have diligently incorporated all the comments and suggestions provided during the review process. I am pleased to see that they have taken the time to address the concerns raised and have made appropriate revisions throughout the paper.

The authors have made substantial improvements to the clarity, methodology, and overall quality of the manuscript. Their responses to the reviewers' comments demonstrate their commitment to enhancing the research and ensuring its accuracy. I appreciate the thoroughness with which they have revised the manuscript, and I believe their efforts have significantly strengthened the paper.

Based on the revised version, I am confident that the manuscript is now suitable for publication. The authors have adequately addressed all the issues raised during the review process, and the paper now meets the high standards expected by our journal.

Thank you for considering my recommendation. I am available for any further clarification or discussion if needed.

Reviewer #4: The revised version incorporates the suggested changes effectively, enhancing the overall clarity and coherence of the content. I am confident that the authors have carefully considered the comments and made appropriate adjustments, which have resulted in a more refined and satisfactory piece of work.

**********

7. PLOS authors have the option to publish the peer review history of their article (what does this mean?). If published, this will include your full peer review and any attached files.

If you choose “no”, your identity will remain anonymous but your review may still be made public.

**Do you want your identity to be public for this peer review?** For information about this choice, including consent withdrawal, please see our Privacy Policy.

Reviewer #3: **Yes: **Idrees Liaqat

Reviewer #4: No

**********

# Editor report: Acceptance letter

**DOI:** 10.1371/journal.pone.0288778.r006

**Authors:** Shujahat Haider Hashmi

10 Jul 2023

PONE-D-22-32398R2 

An analysis of the leverage effect of “Internet +” on the economic spillover of sports industry 

Dear Dr. Yi:

I'm pleased to inform you that your manuscript has been deemed suitable for publication in PLOS ONE. Congratulations! Your manuscript is now with our production department. 

If your institution or institutions have a press office, please let them know about your upcoming paper now to help maximize its impact. If they'll be preparing press materials, please inform our press team within the next 48 hours. Your manuscript will remain under strict press embargo until 2 pm Eastern Time on the date of publication. For more information please contact onepress@plos.org.

If we can help with anything else, please email us at plosone@plos.org. 

Thank you for submitting your work to PLOS ONE and supporting open access. 

Kind regards, 

PLOS ONE Editorial Office Staff

on behalf of

Dr. Shujahat Haider Hashmi 

Academic Editor

PLOS ONE
